# Fibril‐Type Textile Electrodes Enabling Extremely High Areal Capacity through Pseudocapacitive Electroplating onto Chalcogenide Nanoparticle‐Encapsulated Fibrils

**DOI:** 10.1002/advs.202203800

**Published:** 2022-09-25

**Authors:** Woojae Chang, Donghyeon Nam, Seokmin Lee, Younji Ko, Cheong Hoon Kwon, Yongmin Ko, Jinhan Cho

**Affiliations:** ^1^ Department of Chemical and Biological Engineering Korea University 145 Anam‐ro, Seongbuk‐gu Seoul 02841 Republic of Korea; ^2^ Division of Energy Engineering Kangwon National University 346 Jungang‐ro Samcheok 25913 Republic of Korea; ^3^ Division of Energy Technology Daegu Gyeongbuk Institute of Science and Technology (DGIST) 333 Techno Jungang‐daero, Hyeonpung‐eup, Dalseong‐gun Daegu 42988 Republic of Korea; ^4^ KU‐KIST Graduate School of Converging Science and Technology Korea University 145 Anam‐ro, Seongbuk‐gu Seoul 02841 Republic of Korea

**Keywords:** chalcogenide nanoparticles, energy storage, multi‐stacking, pseudocapacitve electroplating, textile pseudocapacitor

## Abstract

Effective incorporation of conductive and energy storage materials into 3D porous textiles plays a pivotal role in developing and designing high‐performance energy storage devices. Here, a fibril‐type textile pseudocapacitor electrode with outstanding capacity, good rate capability, and excellent mechanical stability through controlled interfacial interaction‐induced electroplating is reported. First, tetraoctylammonium bromide‐stabilized copper sulfide nanoparticles (TOABr‐CuS NPs) are uniformly assembled onto cotton textiles. This approach converts insulating textiles to conductive textiles preserving their intrinsically porous structure with an extremely large surface area. For the preparation of textile current collector with bulk metal‐like electrical conductivity, Ni is additionally electroplated onto the CuS NP‐assembled textiles (i.e., Ni‐EPT). Furthermore, a pseudocapacitive NiCo‐layered double hydroxide (LDH) layer is subsequently electroplated onto Ni‐EPT for the cathode. The formed NiCo‐LDH electroplated textiles (i.e., NiCo‐EPT) exhibit a high areal capacitance of 12.2 F cm^−2^ (at 10 mA cm^−2^), good rate performance, and excellent cycling stability. Particularly, the areal capacity of NiCo‐EPT can be further increased through their subsequent stacking. The 3‐stack NiCo‐EPT delivers an unprecedentedly high areal capacitance of 28.8 F cm^−2^ (at 30 mA cm^−2^), which outperforms those of textile‐based pseudocapacitor electrodes reported to date.

## Graphical abstract

Herein, high‐performance pseudocapacitor textile electrodes prepared from chalcogenide nanoparticle assembly‐driven pseudocapacitive electroplating process are introduced. The research motivation is based on the possibility that the effective incorporation of conductive and energy storage materials into 3D porous textiles using favorable interfacial interactions plays a pivotal role in developing high‐performance energy storage devices.

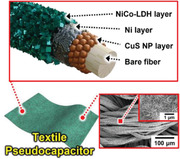

## 1 Introduction

Pseudocapacitors, which can compensate for the low energy density of conventional electrical double‐layer capacitors (EDLCs), have been recognized as a promising power source.^[^ ^1^, ^2^, ^3^, ^4^, ^5^ ^]^ Particularly, the recent rapid progress in portable electronics and explosive growth in their use have strongly stimulated the development of pseudocapacitors enabling higher areal capacitance and energy density while maintaining good rate performance and high operational stability. However, most electrodes for energy storage applications have been prepared in the form of thin film configurations on nonporous metal foils, which have an intrinsic limitation on increasing the thickness (or loading amount) of active materials due to the increased charge resistance for thicker electrodes.^[^ ^6^, ^7^, ^8^ ^]^ To address these problems, several different approaches have been proposed, which have mainly focused on the use of unique active materials, the preparation of porous current collectors, and/or the design of optimal electrode structures.

Recently, pseudocapacitor electrodes based on inexpensive, flexible, and conductive textile current collectors (TCCs) have emerged as notable candidates that can resolve the abovementioned drawbacks of pseudocapacitor electrodes with a limited 2D surface area.^[^ ^9^, ^10^, ^11^, ^12^, ^13^, ^14^, ^15^, ^16^, ^17^ ^]^ Particularly, the 3D porous structure of TCCs composed of numerous interconnected fibrils not only provides a large surface area for a high loading amount of active material per unit area but also induces facile ion diffusion into active materials.^[^ ^16^, ^18^, ^19^, ^20^, ^21^ ^]^ However, for these insulating fibril‐type textiles to be effectively used as 3D porous current collectors, they should be first converted to conductive textiles through uniform coating of conductive materials. A variety of conductive materials, including carbon nanotubes (CNTs) and metal nanowires (NWs), have been incorporated into textiles through conventional physical adsorption processes.^[^ ^22^, ^23^, ^24^ ^]^ However, these TCCs exhibit much lower electrical conductivity (sheet resistance > 10 Ω sq^−1^ and electrical conductivity < 200 S cm^−1^) than bulk metal foil because of the numerous contact resistances existing between adjacent conductive materials (CNTs and NWs), which increases the charge transfer resistance between TCCs and active materials as well as the internal resistance of electrodes. Although it has been reported that TCCs with an extremely low sheet resistance of approximately 0.5 Ω sq^−1^ can be prepared through multilayer films composed of dozens of Au nanoparticles (NPs),^[^ ^16^, ^25^, ^26^, ^27^, ^28^ ^]^ the use of such noble metal NPs can significantly restrict the commercial availability.

Electroless deposition method through the reduction of metal ions from a precursor solution (i.e., chemical reduction deposition) has also been employed to fabricate high‐performance TCCs.^[^ ^29^, ^30^ ^]^ Although electroless‐deposited metal textiles can possess a higher electrical conductivity compared to carbon material‐incorporated textiles or carbon cloths, they still exhibit a lower electrical conductivity than bulk metal foil due to the presence of organic impurities in the metal precursors. At the same time, their nonuniform metal coating decreases the high specific surface area of the pristine textile and limits the charge transfer efficiency. As a result, the reported approaches have much difficulty in fully utilizing the advantages of TCCs.

Although TCCs can be successfully prepared by some innovative approaches, it should be noted that the coating quality of active materials (i.e., pseudocapacitive components) on TCCs also has a significant effect on the overall performance of electrodes. Generally, an active slurry composed of transition metal oxides and additives (i.e., conductive carbon materials and insulating polymer binders) is deposited onto TCCs for completion of pseudocapacitor electrodes.^[^ ^14^, ^31^, ^32^ ^]^ However, the use of a randomly mixed active slurry imposes a limit on the uniform deposition of the active materials onto all regions of TCCs due to the unfavorable interfacial interactions among the respective components. Particularly, the introduced polymer binders impose considerable restrictions on the charge transfer at the TCC/active material and active material/active material interfaces.

As an alternative, pseudocapacitive materials have been directly deposited onto conductive TCCs through hydrothermal and/or electroless deposition without the aid of polymer binders.^[^^]^ However, in most cases, the abovementioned deposition processes (hydrothermal or electroless deposition) have been performed on TCCs without deep consideration of interfacial interactions between TCCs and pseudocapacitive components. In this view, one of the most challenging approaches is to perfectly convert all insulating fibrils within textiles to bulk metal‐like conductive fibrils (for a current collector) through conformal coating of conductive seeds. Furthermore, the high‐energy pseudocapacitive components should be uniformly and densely coated on the formed fibril conductors through a favorable interfacial interaction‐mediated approach.

**Scheme 1:**
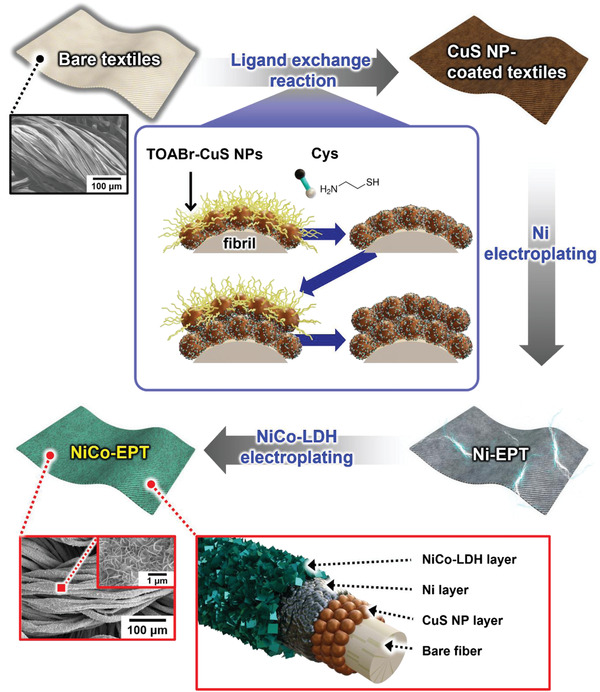
Schematic representation of the preparation of NiCo‐EPT as pseudocapacitor electrodes through a chalcogenide NP assembly‐induced electroplating process.

Herein, we introduce high‐performance pseudocapacitor textile electrodes prepared from chalcogenide (copper sulfide; CuS) NP assembly‐driven pseudocapacitive electroplating (**Scheme** **1**). Our interfacial interaction‐mediated approach is characterized by the fact that all the interfaces within the textile‐based pseudocapacitive electrodes are covalent‐ and metallic bonded with each other, and additionally their nanosized structures further increase the specific surface area of pristine textiles. These favorable interfacial interactions and structural uniqueness significantly enhance the energy storage performance of pseudocapacitive textiles showing high mechanical flexibility. For this study, tetraoctylammonium bromide (TOABr)‐stabilized copper sulfide NPs (i.e., TOABr‐CuS NPs) were newly synthesized and layer‐by‐layer (LbL) assembled with amine (‐NH_2_)/thiol (‐SH)‐functionalized cysteamine (Cys) onto highly porous cotton textiles. However, due to the relatively low intrinsic conductivity of CuS (the conductivity of bulk CuS ≈2 × 10^3^ S cm^−1^ at room temperature)^[^ ^34^ ^]^ and the numerous contact resistances at CuS NP‐NP interfaces, Ni electroplating was additionally carried out on the (TOABr‐CuS NP/Cys)*_n_*‐coated textiles to overcome the relatively low electrical conductivity. In this case, the Ni electroplated textiles (i.e., Ni‐EPT) realized bulk metal‐like conductivity (sheet resistance ≈0.06 Ω sq^−1^). Particularly, the high affinity between the electroplated Ni layer and the NH_2_ groups of the outermost Cys layer as well as the highly uniform adsorption of conductive CuS NPs onto cotton fibrils induced conformal coating of the robust, electroplated Ni layer onto all regions ranging from the exterior to the interior of the textiles.

Based on these Ni electroplated TCCs (i.e., Ni‐EPT), pseudocapacitive NiCo layered double hydroxide (LDH) was sequentially electroplated for the textile cathode (i.e., NiCo‐EPT). Generally, it has been reported that Ni‐ or Co‐ based metal oxides/hydroxides show much higher theoretical capacitances (i.e., 2358 F g^−1^ for Ni(OH)_2_, 3108 F g^−1^ for Co(OH)_2_) than conventional pseudocapacitive materials such as MnOx.^[^ ^35^, ^36^, ^37^, ^38^, ^39^ ^]^ Moreover, these bimetallic metal hydroxides can exhibit higher theoretical capacity due to their multiple redox activity (Ni^2+^/Ni^3+^ and Co^2+^/Co^3+^) as well as relatively high electrical conductivity (0.1–100 S cm^−1^).^[^ ^40^, ^41^, ^42^, ^43^, ^44^, ^45^ ^]^ Therefore, in the case of NiCo‐EPT, the highly porous structure of the fibril‐type textile was well preserved along with high electrochemical activity, extremely low sheet resistance (≈0.06 Ω sq^−1^), and high mechanical flexibility. Particularly, when the mass loading of electroplated NiCo‐LDH was increased to 5.12 mg cm^−2^, the areal capacitance of NiCo‐EPT was significantly increased to 12.2 F cm^−2^, which could be further enhanced by simple multi‐stacking of NiCo‐EPTs (i.e., the areal capacitance of 3‐stacked NiCo‐EPT ≈28.8 F cm^−2^). This high areal capacitance outperformed those of previously reported textile electrodes. Considering that the interfacial interactions and structural quality within electrodes as well as the intrinsic electrochemical properties of active materials have a dominant effect on the overall energy performance of electrodes, our approach can provide an important basis for developing and designing a variety of high‐performance energy storage electrodes, including pseudocapacitors.

## 2 Results and Discussion

### 2.1 Conductive Textile Using LbL‐Assembled CuS NP Multilayers

**Figure 1:**
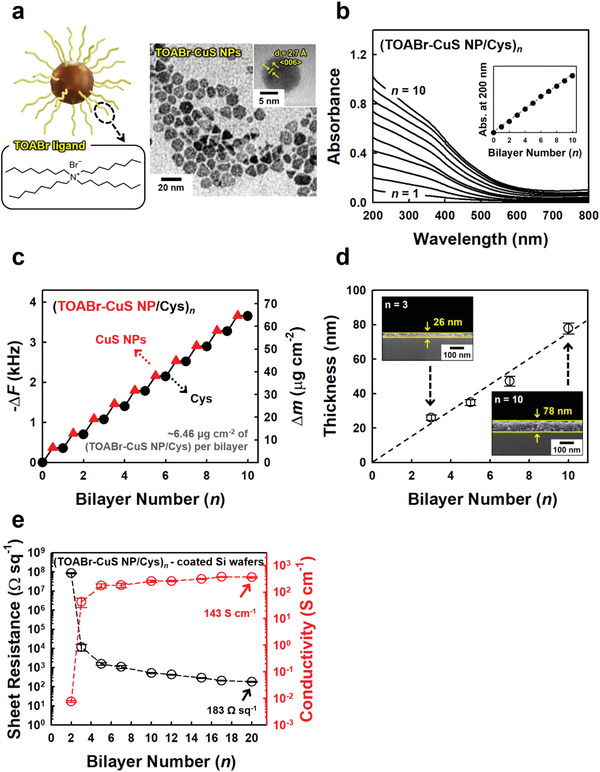
a) Molecular structure of the TOABr ligand, and TEM image of TOABr‐CuS NPs with a diameter of approximately 8 nm. The inset in the TEM image shows a lattice spacing (*d*) of ≈2.7 Å corresponding to the (006) plane of CuS. b) UV–vis absorbance spectra of (TOABr‐CuS NP/Cys)*_n_* multilayers coated onto quartz glasses as a function of the bilayer number (*n*). The inset shows the absorbance at a wavelength of 200 nm, indicating linear growth of multilayers. c) Frequency (−Δ*F*, left axis) and mass change (Δ*m*, right axis) of (TOABr‐CuS NP/Cys)*_n_* multilayers with increasing bilayer number obtained from QCM analysis. d) Thickness change of (TOABr‐CuS NP/Cys)*_n_* multilayers on Si wafers as a function of the bilayer number (*n*), measured from the cross‐sectional FE‐SEM images (inset). e) Sheet resistance (left axis) and electrical conductivity (right axis) of (TOABr‐CuS NP/Cys)*_n_* multilayers on Si wafers as a function of the bilayer number (*n*).

To prepare conductive cotton textiles, TOABr‐CuS NPs with a diameter of approximately 8 nm as non‐noble metals, were first synthesized in nonpolar media (**Figure** **1**a; Figure S1, Supporting Information). These bulky TOABr ligands (M_w_ ≈546 g mol^−1^) loosely bound to the surface of CuS NPs could be easily replaced by small Cys molecular ligands (M_w_ ≈77 g mol^−1^) in organic media due to the higher affinity between the NH_2_/SH groups of Cys and the bare surface of CuS NPs. This phenomenon is very important in that TOABr‐CuS NPs can be successively assembled with Cys molecules via repetitive ligand exchange reactions during LbL deposition and additionally the separation distance between vertically adjacent CuS NP layers can be considerably shortened by Cys molecular ligands, resulting in a significant decrease in the contact resistance between neighboring CuS NPs.

To confirm these possibilities, the adsorption mechanism of LbL‐assembled (TOABr‐CuS NP/Cys)*_n_* multilayers was further investigated using Fourier transform infrared (FT‐IR) spectroscopy as a function of the bilayer number (*n*) (Figure S2, Supporting Information). As shown in Figure S2, Supporting Information, the deposition of TOABr‐CuS NPs onto the Cys coated substrate (i.e., *n* = 0.5) generated C—H stretching peaks from the bulky alkyl chains of TOABr ligands at 2850–2950 cm^−1^. When Cys ligands were further deposited onto the TOABr‐CuS NP‐coated substrate (*n* = 1.0), the C—H stretching peak significantly decreased. Therefore, the alternating deposition of TOABr‐CuS NPs and Cys ligands caused repeated generation and disappearance of the C—H stretching peaks from the TOABr ligands when the outermost layer was periodically changed from TOABr‐CuS NPs to Cys and vice versa. These results evidently implied that LbL‐assembled (TOABr‐CuS NP/Cys)*_n_* multilayers could be prepared using the ligand exchange reaction between bulky TOABr and small Cys ligands.

Based on these results, we investigated the adsorption behavior of LbL‐assembled (TOABr‐CuS NP/Cys)*_n_* multilayers on various flat substrates using UV–vis spectroscopy, a quartz crystal microbalance (QCM), and field‐emission scanning electron microscopy (FE‐SEM) (Figure 1b–d). First, from the UV–vis absorbance data, the LbL‐assembled multilayers qualitatively exhibited linear growth with increasing bilayer number, implying the formation of complementary interactions between TOABr‐CuS NPs and Cys and vertical growth of multilayers (Figure 1b). We also quantitatively examined the loading amount of the (TOABr‐CuS NP/Cys)*_n_* multilayers using QCM electrodes. Figure 1c shows the frequency change (−Δ*F*) of alternatively deposited TOABr‐CuS NPs and Cys ligands, resulting in approximately 365 ± 7 Hz per (TOABr‐CuS NP/Cys) bilayer (389 Hz for TOABr‐CuS NPs and −24 Hz for Cys). Converting −Δ*F* into mass change (Δ*m*) using the Sauerbrey equation, Δ*m* corresponds to 6.46 µg cm^−2^ per bilayer (6.88 µg cm^−2^ for TOABr‐CuS NPs and −0.42 µg cm^−2^ for Cys) (more detailed information is given in the Experimental Section). Particularly, a slight decrease in the frequency or mass change during the deposition of Cys implies that the bulky native TOABr ligands bound to the CuS NPs were replaced by smaller Cys molecules. Additionally, these results evidently demonstrate that only one Cys layer exists between vertically adjacent CuS NP layers after LbL deposition. Although we previously reported that NH_2_‐rich small molecular ligands such as tris(2‐aminoethyl)amine (TREN) or diethylenetriamine (DETA) could be used as effective organics for ligand exchange‐induced LbL assembly of TOABr‐Au NPs, the TOABr‐CuS NPs were easily dissociated in these solutions due to the formation of strong complexes between Cu ions and the organics (i.e., TREN or DETA) with high‐density NH_2_ groups per carbon. As a result, CuS NPs could not be easily LbL‐assembled with TREN or DETA ligands (Figure S3, Supporting Information). In contrast, it should be noted that Cys ligands with only one NH_2_ group and one SH group can stably induce the adsorption of TOABr‐CuS NPs. By increasing the bilayer number from 3 to 10, the total film thicknesses of the (TOABr‐CuS NP/Cys)*_n_* multilayers increased from approximately 26 to 78 nm, forming a highly uniform and nanoporous structure (Figure 1d; Figure S4, Supporting Information).

Based on these results, the electrical properties of the (TOABr‐CuS NP/Cys)*_n_* multilayer film on Si wafers were examined as a function of the bilayer number (*n*) (Figure 1e). As the bilayer number increased from 2 to 20, the sheet resistance of the multilayer film notably decreased from approximately 8.7 × 10^7^ to 183 Ω sq^−1^, and additionally, the electrical conductivity (*σ*) increased from 7.7 × 10^−3^ to 365 S cm^−1^ without any additional chemical, physical, and/or thermal treatments. Although the electrical conductivity of the LbL‐assembled CuS NP multilayers was much inferior to that of bulk pure Cu (i.e., the electrical conductivity of bulk Cu ≈5.96 × 10^5^ S cm^−1^ at 20 °C), it should be noted that preparing conductive films from spherical‐type CuS‐based NPs with numerous contact resistances at room temperature is very challenging, which, to our knowledge, have rarely been reported by other research groups.

**Figure 2:**
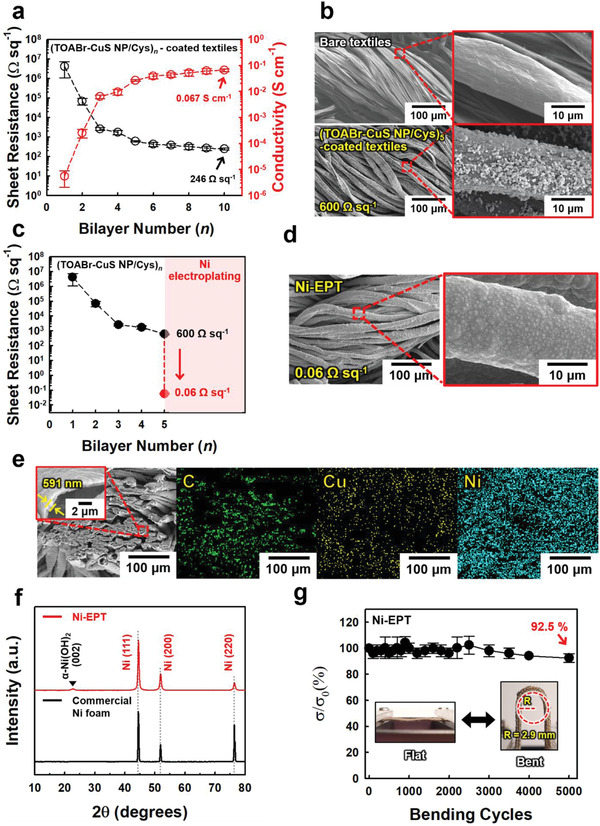
a) Electrical properties of (TOABr‐CuS NP/Cys)*_n_*‐coated textiles with increasing bilayer number (*n*). b) Planar FE‐SEM images of bare textiles (top) and (TOABr‐CuS NP/Cys)_5_‐coated textiles (bottom) at different magnifications. c) Electrical properties after the Ni electroplating process at the condition of 250 mA cm^−2^ for 10 min. d) Planar FE‐SEM images at different magnifications, and e) cross‐sectional FE‐SEM and corresponding EDX mapping images of Ni‐EPT. f) XRD patterns of Ni‐EPT and commercial Ni foams. g) Relative electrical conductivity (*σ*/*σ*_0_) of Ni‐EPT as a function of the bending cycles (bending radius of ≈2.9 mm).

On the basis of these results, our approach was expanded to a 3D porous cotton textile composed of hydroxyl (OH) group‐functionalized cellulose fibrils. Similarly, TOABr‐CuS NPs were assembled onto the Cys‐coated textile through the ligand exchange reaction between TOABr and Cys ligands. With increasing bilayer number of (TOABr‐CuS NP/Cys)*_n_* multilayers from 1 to 10, the sheet resistance of the textiles decreased from ≈10^7^ to 246 Ω sq^−1^, and their electrical conductivity increased from ≈10^−5^ to 6.7 × 10^−2^ S cm^−1^ (herein, the measured electrical conductivity was calculated including the thickness of 620 µm‐thick cotton textiles) (**Figure** **2**a). The formed multilayers were densely deposited onto all fibrils within cotton textiles without the blocking‐up phenomena of porosity (Figure 2b). Additionally, (TOABr‐CuS NP/Cys)_5_‐coated textiles exhibited stable mechanical and electrical properties under repeated external stimuli (Figure S5, Supporting Information).

### 2.2 Highly Conductive TCCs Using Ni Electroplating

However, it should be noted that it is difficult to prepare highly conductive CuS NP‐coated textiles with extremely low sheet resistance (< 10^−1^ Ω sq^−1^) through subsequent LbL deposition of CuS NPs due to the intrinsically low electrical conductivity. To overcome this critical problem and further improve the process efficiency, Ni electroplating was additionally performed on the CuS NP‐coated textiles. In this case, we expected that the sheet resistance of ≈600 Ω sq^−1^ measured from the 5 bilayer (TOABr‐CuS NP/Cys)‐coated textiles would be sufficiently suitable for Ni electroplating. To confirm this possibility, the (TOABr‐CuS NP/Cys)_5_‐coated textiles were electroplated, and the change in the sheet resistance was observed at various external current densities and electroplating times (Figure 2c; Figure S6, Supporting Information). As the current density and electroplating time increased from 50 mA cm^−2^ and 2 min to 250 mA cm^−2^ and 10 min, the sheet resistance of the Ni electroplated textiles decreased to 0.06 Ω sq^−1^.

Based on these results, Ni electroplating condition of 250 mA cm^−2^ for 10 min was used to produce Ni‐EPT. As confirmed by FE‐SEM (Figure 2d) and energy‐dispersive X‐ray (EDX) mapping (Figure 2e) images of Ni‐EPT, the electroplated Ni components were uniformly deposited onto all the fibrils from the outermost surface to the central region of the textiles without Ni agglomeration, with a thickness of 591 nm. This was entirely attributed to both the formation of conformal conductive seed layers (i.e., CuS NP multilayers) on cotton fibrils and the favorable interfacial interactions between the outermost Cys layer and the Ni precursor ions.

The crystal structure of the Ni‐EPT layer was analyzed using X‐ray diffraction (XRD) analysis. The XRD pattern of Ni‐EPT exhibited (111), (200), and (220) reflection peaks located at 44.6°, 51.8°, and 76.4° respectively, which are attributed to the characteristic peaks of face‐centered cubic (fcc) Ni.^[^ ^46^, ^47^ ^]^ Additionally, a minor (002) peak assigned to *α*‐Ni(OH)_2_ was observed due to partial surface oxidation.^[^ ^30^ ^]^ These results were nearly consistent with that of commercial Ni foams (Figure 2f), implying that insulating cotton textiles were converted to bulk Ni‐like conductive textiles through the LbL assembly and Ni electroplating processes. Furthermore, this conductive layer preserved an extremely low sheet resistance, retaining 92.5% of the initial conductivity, after 5000 cycles of repetitive mechanical bending tests (Figure 2g). These results imply that Ni‐EPT formed through our interfacial interaction‐mediated approach could be used as bulk metal‐like conductive TCCs with excellent mechanical stability and electrical stability.

Although TCCs can also be prepared by electroless deposition method using chemical reduction of Ni precursor ions (i.e., Ni‐CRT), it is difficult to produce highly uniform deposition and maintain porous structures due to the inadequate control of the interfacial interactions between the textile fibrils and the introduced Ni components (Figure S7, Supporting Information). That is, cationic Ni ions are sparsely adsorbed onto the textiles during the deposition process due to the strong electrostatic repulsion occurring among the same charge Ni ions. With the aid of a reducing agent, Ni ions are chemically reduced to Ni seeds with low packing density, and Ni cations are sustainedly incorporated into the Ni seeds. As a result, the selective growth of Ni seeds (i.e., nucleation growth) induces nonuniform Ni deposition onto the cotton textiles. Particularly, because of the presence of organic impurities, the electroless‐deposited Ni layer imposes a limit on securing a sheet resistance value (≈5 Ω sq^−1^) comparable to that of Ni‐EPT. These phenomena generated a crystal structure of the electroless Ni layer that was different from that of Ni‐EPT or bulk Ni foams.

Specifically, the XRD pattern of Ni‐CRT exhibited the characteristics of the (001) and (002) peaks for *α*‐Ni(OH)_2_ and (001) and (101) peaks for *β*‐Ni(OH)_2_ as well as the (111) peak for fcc‐Ni (Figure S8, Supporting Information).^[^ ^30^ ^]^ Additionally, the dominant peak of *α*‐Ni(OH)_2_ instead of thermodynamically stable *β*‐Ni(OH)_2_ implied that electroless deposition strongly induced the metastable phase because of the relatively low reduction power and organic impurities. Furthermore, Ni‐CRT showed rapid degradation of the electrical conductivity under repeated external stimuli (Figure S9, Supporting Information), which was in stark contrast to Ni‐EPT.

### 2.3 Pseudocapacitive Textiles Using Pseudocapacitive Electroplating

**Figure 3:**
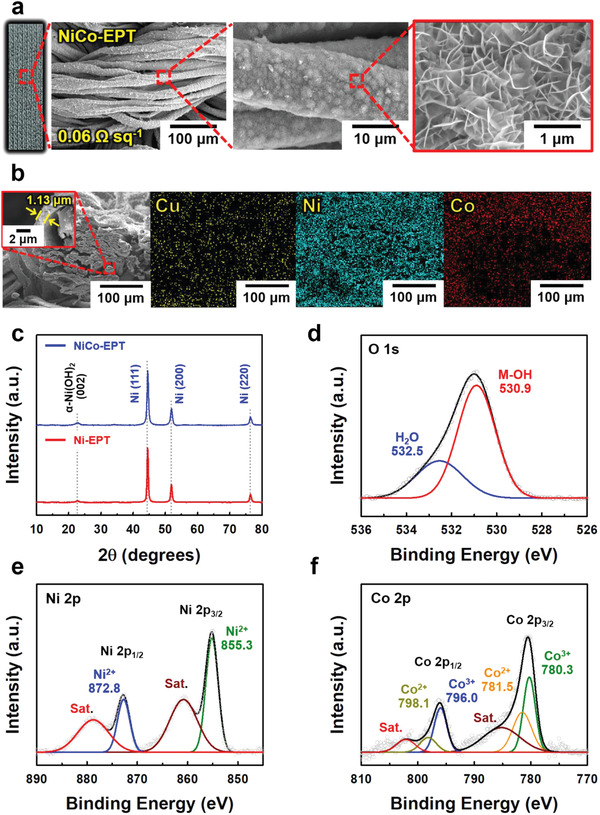
a) Photographic/planar FE‐SEM images at different magnifications, and b) cross‐sectional FE‐SEM and corresponding EDX mapping images of NiCo‐EPT electrodes. c) XRD pattern of NiCo‐EPT in comparison with that of Ni‐EPT. High‐resolution XPS spectra of d) O 1s, e) Ni 2p, and f) Co 2p of the NiCo‐EPT electrodes.

To prepare a pseudocapacitive textile cathode with enhanced energy storage performance, a NiCo‐LDH material exhibiting high theoretical capacity was additionally electroplated onto the Ni‐EPT current collector at a current density of 3 mA cm^−2^ for different electroplating times (i.e., NiCo‐t) (more details in the Experimental Section). In this case, NiCo‐LDHs were uniformly electroplated onto the respective fibrils with increasing the electroplating time from 15 to 60 min, which was in stark contrast with nonuniform NiCo‐LDHs formed at a current density of 5 mA cm^−2^ (Figures S10 and S11, Supporting Information). Additionally, their mass loading increased almost linearly from 1.27 to 5.12 mg cm^−2^ according to the corresponding electroplating time (i.e., from 15 to 60 min) (Figure S12, Supporting Information). Particularly, in the case of an electroplating time of 60 min (NiCo‐60, especially designated as NiCo‐EPT), the formed NiCo‐LDH layer exhibited a well‐defined structure with a thickness of 539 nm (i.e., calculated from both the total thickness of 1.13 µm and the Ni layer thickness of 591 nm) and was homogeneously deposited onto all fibrils within the textile without any block‐up of porosity despite the relatively high mass loading (5.12 mg cm^−2^), which was confirmed by FE‐SEM and EDX mapping images (**Figure** **3**a,b). Additionally, this NiCo‐EPT exhibited a considerably low sheet resistance of approximately 0.06 Ω sq^−1^, similar to that of Ni‐EPT, and maintained a relatively stable electrical conductivity, as confirmed by the mechanical stability test (Figure S13, Supporting Information).

From the high‐resolution transmission electron microscopy (HR‐TEM) and EDX mapping images, no ordered crystal structure was found in the homogeneously mixed layer of NiCo‐LDH layer (Figure S14, Supporting Information). Additionally, as shown in Figure 3c, the XRD pattern of NiCo‐EPT exhibited reflection peaks of (111), (200), and (220) originating from Ni‐EPT, implying that the NiCo‐LDH layer possessed an amorphous phase. Especially, it has been reported that the amorphous structure of NiCo‐based components, which possess many defects and long‐range disordered characteristics, can provide an easier pathway for intercalation/deintercalation of charges in the electrolyte.^[^ ^48^ ^]^ As a result, this amorphous structure of NiCo‐LDH can favor the redox reaction, resulting in the enhancement of the charge transfer rate of our textile electrodes. The chemical states of the bonded elements and valence state of NiCo‐EPT were also investigated using X‐ray photoelectron spectroscopy (XPS) analysis. In the case of the O 1s spectrum, the main peak at 530.9 eV was caused by metal hydroxyl groups (Ni—OH and Co—OH), and the other peak at 532.5 eV originated from the adsorbed water molecules (Figure 3d).^[^ ^48^, ^49^ ^]^ Peaks at binding energies of 855.3 (for Ni 2p_3/2_) and 872.8 eV (for Ni 2p_1/2_), as well as two satellite peaks at 860.9 and 878.8 eV, were observed in the high‐resolution spectra of Ni 2p (Figure 3e).^[^ ^49^, ^50^ ^]^ The deconvoluted XPS spectrum of Co 2p indicated the presence of mixed Co^2+^ and Co^3+^ (Figure 3f).^[^ ^49^, ^51^ ^]^ Additionally, the Ni/Co atomic ratio on the surface of the formed 539 nm‐thick NiCo‐LDH layer was estimated to be approximately 1.32:1 from the XPS spectrum areas.

The specific surface area of NiCo‐EPT and NiCo‐LDH electroplated Ni foam (NiCo/Ni foam) was further investigated using mercury porosimetry technique. As a result, the measured specific surface area of NiCo‐EPT was approximately 5.539 m^2^ g^−1^, which was larger than that of NiCo/Ni foam (≈3.768 m^2^ g^−1^) (Figure S15, Supporting Information). This result suggested the possibility that the areal electrochemical performance of NiCo‐EPT could significantly outperform that of commercial Ni foam‐based electrodes.

**Figure 4:**
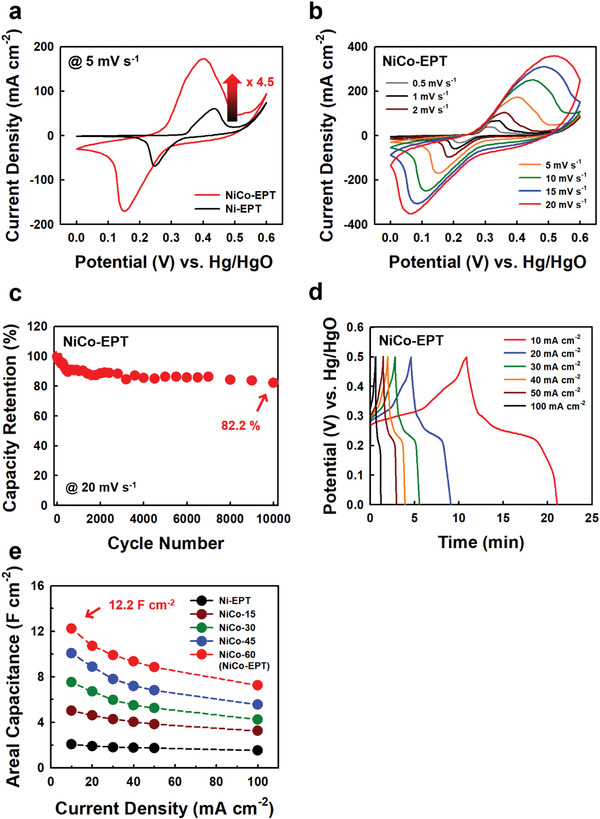
a) Comparison of CV curves with NiCo‐EPT and Ni‐EPT electrodes at a scan rate of 5 mV s^−1^. b) CV curves of NiCo‐EPT electrodes at various scan rates in the range of 0.5 to 20 mV s^−1^. c) Capacity retention of NiCo‐EPT electrodes at a scan rate of 20 mV s^−1^ during 10 000 cycles. d) GCD curves of NiCo‐EPT electrodes at various current densities in the range of 10 to 100 mA cm^−2^. e) Areal capacitance of NiCo‐t electrodes with various electroplating times (15–60 min) at a constant current density (3 mA cm^−2^) in comparison with that of Ni‐EPT electrodes for various current densities from 10 to 100 mA cm^−2^.

Based on these results, the electrochemical performance of NiCo‐EPT (obtained from 60 min‐electroplating time) as a cathode for pseudocapacitors was evaluated using a three‐electrode cell configuration in a 6 m KOH electrolyte. Cyclic voltammetry (CV) scans of the electrodes were measured at a scan rate of 5 mV s^−1^ (**Figure** **4**a). First, the integrated CV area of NiCo‐EPT in the potential range of 0−0.6 V (vs the Hg/HgO reference electrode) was much larger than that of bare Ni‐EPT, suggesting that the overall capacitance of NiCo‐EPT was mainly induced by that of the electroplated NiCo‐LDH layer. Additionally, NiCo‐EPT electrodes displayed a pair of redox peaks originating from the reversible Faradaic reaction during electrochemical measurements. These electrochemical reactions are as follows:^[^ ^52^, ^53^ ^]^

(1) $$\mathrm{Ni}{\left(\mathrm{OH}\right)}_{2}+OH^{-}↔\mathrm{NiOOH}+H_{2}O+e^{-}$$

(2) $$\mathrm{Co}{\left(\mathrm{OH}\right)}_{2}+OH^{-}↔\mathrm{CoOOH}+H_{2}O+e^{-}$$

(3) $$\mathrm{CoOOH}+OH^{-}↔{\mathrm{CoO}}_{2}+H_{2}O+e^{-}$$

The integrated area of the CV curves at a scan rate of 5 mV s^−1^ gradually increased as the NiCo‐LDH electroplating time increased, implying that the capacitance of the electrode can be controlled by adjusting the electroplating time (Figure S16, Supporting Information). Especially, the CV curve of 15 min NiCo‐LDH electroplated textiles (i.e., NiCo‐15) exhibited a reduction peak at 0.45 V due to the insufficient coating of the current collector, which led to partial expression of Ni‐EPT. All CV curves of NiCo‐EPT displayed an evident redox pair without notable deformation in the shape at different scan rates ranging from 0.5 to 20 mV s^−1^ (Figure 4b; Figure S17, Supporting information). Particularly, the integrated CV areas of Ni‐EPT and NiCo‐EPT were much larger than those of commercial Ni foam‐based electrodes (i.e., bare Ni foam and NiCo/Ni foam) (Figure S18, Supporting Information). These results evidently implied that our electrodes are more electrochemically active and have larger surface areas than commercial Ni foam‐based electrodes. Additionally, NiCo‐EPT retained 82.2% of its initial capacitance after 10 000 cycles (at a scan rate of 20 mV s^−1^), while maintaining the surface morphology without delamination and/or crack of the NiCo‐LDH layer (Figure 4c; Figure S19, Supporting Information).

We analyzed the CV measurements to comprehend the capacitive charge storage characteristics of NiCo‐EPT electrodes. Generally, the charge storage behaviors can be broadly classified into two types: 1) a surface‐controlled charge storage mechanisms including non‐Faradaic electric double layer capacitance (EDLC) and fast Faradaic pseudocapacitive reaction and 2) a diffusion‐controlled charge storage mechanisms via intercalations of the charge carriers.^[^ ^2^, ^54^ ^]^ The peak currents at anodic and cathodic sweeps at different scan rates can be expressed by the following equation:

(4) $$i=av^{b}$$

where *i* and *v* stands for current and scan rate, respectively and additionally, *a* and *b* are variable coefficients. The surface‐ and diffusion‐controlled charge storage processes are proportional to *v* (*b* = 1) and *v* ^1/2^ (*b* = 0.5), respectively. As depicted in Figure S20a, Supporting Information, the *b*‐values were obtained from the slope of log‐log plots of peak current versus scan rate at both anodic and cathodic sweeps. We further discussed the contribution ratio of charge storage between the surface‐ and diffusion‐controlled reactions by the following relation:^[^ ^2^, ^55^ ^]^

(5) $$i\left(V\right)=k_{1}v+k_{2}v^{1/2}$$

where *i*(*V*) and *v* correspond to CV current at certain potential and scan rate, respectively. *k*_1_ *v* and *k*_2_ *v* ^1/2^ denote surface‐ and diffusion‐controlled processes, respectively and *k*_1_ and *k*_2_ can be obtained from the slope and y‐intercept values in the following equation:

(6) $$\frac{i\left(V\right)}{v^{1/2}}=k_{1}v^{1/2}+k_{2}$$

Based on Equations (5) and (6), Figure S20b, Supporting Information, depicts the contribution of surface‐controlled charge storage processes of NiCo‐EPT electrode at 5 mV s^−1^, accounting for 66% of the total charge. As shown in Figure S20c, Supporting Information, as the scan rate increased, the contribution of diffusion‐controlled charge storage processes decreased due to the insufficient reaction time for full activation of diffusion‐based slow Faradaic storage processes such as ion intercalations.

The galvanostatic charge‐discharge (GCD) profiles for NiCo‐EPT were also recorded to practically evaluate the charge storage capabilities at various current densities ranging from 10 to 100 mA cm^−2^ (Figure 4d). In this case, all GCD curves with a nonlinear profile, indicating the typical pseudocapacitive behavior,^[^ ^56^, ^57^ ^]^ exhibited good rate capability. Particularly, the areal capacitances of NiCo‐EPT at current densities of 10 and 100 mA cm^−2^ were estimated to be approximately 12.2 and 7.2 F cm^−2^, respectively, demonstrating the relatively good rate capability, and also exhibited a high Coulombic efficiency of 93.7% and 98.3% (Figure S21, Supporting Information). Additionally, NiCo‐EPT retained 70.8% of its initial capacitance after 5000 cycles of GCD measurement at a current density of 150 mA cm^−2^ (Figure S22, Supporting Information). Furthermore, it should be noted that the electroplating time of NiCo‐LDH was directly related to the areal capacitance of NiCo‐EPT (Figure 4e; Figure S23, Supporting Information). Specifically, with increasing NiCo‐LDH electroplating time from 15 to 60 min, the areal capacitances of NiCo‐t electrodes at 10 mA cm^−2^ increased from 5.0 to 12.2 F cm^−2^, which also corresponded to specific capacitance of 2383 F g^−1^ and volumetric capacitance of 122 F cm^−3^.

To further investigate the electrochemical behavior of the NiCo‐t electrodes, electrochemical impedance spectroscopy (EIS) measurements were carried out in the frequency range from 10^5^ to 0.1 Hz (Figure S24, Supporting Information). Although these electrodes revealed typical mass loading (i.e., electroplating time)‐dependent pseudocapacitive trends in the low‐frequency region (Warburg impedance), they exhibited no meaningful difference in the equivalent series resistance (*R*_s_) and charge transfer resistance (*R*_ct_) even with a significant increase in the areal mass loading. Additionally, only slight changes in *R*_s_ value (from 0.89 to 1.99 Ω cm^2^) and Warburg impedance of NiCo‐EPT were observed after the electrochemical stability test (i.e., after 10 000 cycles) (Figure S25, Supporting Information). These good electrochemical performances of NiCo‐EPT could be realized by the relatively high electrical conductivity of NiCo‐LDHs as well as the highly uniform fibril structure formed by favorable interfacial interactions between neighboring components (i.e., high affinities at interfaces of NiCo‐LDH/Ni and Ni/conductive seed‐coated textile). As a result, the electrical and structural characteristics of NiCo‐EPT could induce high energy storage performance.

To gain further insight into the effect of the electrical conductivity of TCCs on the electrochemical performance of NiCo‐EPT, the NiCo‐LDH layer was electroplated onto the Ni‐textile with low electrical conductivity of ≈20 Ω sq^−1^ (i.e., L‐NiCo‐EPT), and the electrochemical properties of the resulting L‐NiCo‐EPT were additionally investigated (Figure S26, Supporting Information). In this case, the L‐NiCo‐EPT showed a much smaller integrated CV area with a broad redox pair and large peak separation (Δ*E*_p_) than NiCo‐EPT, indicating a large overpotential due to sluggish redox kinetics. Additionally, the Nyquist plots demonstrated that the L‐NiCo‐EPT exhibited higher internal resistance (*R*_s_ ≈3.85 Ω cm^−2^ and *R*_ct_ ≈0.83 Ω cm^−2^) and lower Warburg impedance than the NiCo‐EPT. These results clearly demonstrate that the electrical conductivity of the current collector (i.e., TCC) greatly affects the electrochemical performance of the pseudocapacitor electrodes.

Another notable benefit of our approach is that the areal capacitance of NiCo‐EPT can be more significantly enhanced through a simple multi‐stacking method. To prove this effect, the electrochemical performance of multi‐stacked NiCo‐EPT was investigated by CV and GCD at various sweep rates (Figure S27, Supporting Information). With increasing the stacking number up to 3, the current levels and integrated CV area of multi‐stacked NiCo‐EPT gradually increased, implying an increase in the charge storage capability. These phenomena could also be confirmed by the increased discharge time at a current density of 30 mA cm^−2^, as shown in the GCD profiles of Figure S27, Supporting Information. Based on these discharge curves (at 30 mA cm^−2^), the areal capacitances were measured to be approximately 19.4 F cm^−2^ for the 2‐stack and 28.8 F cm^−2^ (1875 F g^−1^ and 288 F cm^−3^) for the 3‐stack, respectively, outperforming the areal capacitances of previously reported textile electrodes (Table S1, Supporting Information).^[^ ^50^, ^51^, ^53^, ^58^, ^59^, ^60^, ^61^, ^62^ ^]^ More importantly, these multi‐stacked NiCo‐EPTs still delivered high areal capacitances of 13.6 (for the 2‐stack) and 20.4 F cm^−2^ (for the 3‐stack), respectively, even at a high current density of 100 mA cm^−2^, suggesting excellent rate performance (Figure S28, Supporting Information). Given that a significant increase in the electrode thickness (i.e., an increase in the mass loading of pseudocapacitive active materials per unit area) can inevitably disturb facile charge transport kinetics,^[^ ^5^, ^63^ ^]^ our approach is very efficient in obtaining pseudocapacitive textile electrodes with high areal capacitance, good rate capability, and large area (Figure S29, Supporting Information).

### 2.4 Electrochemical Performance of Asymmetric Full‐Cell Pseudocapacitors

**Figure 5:**
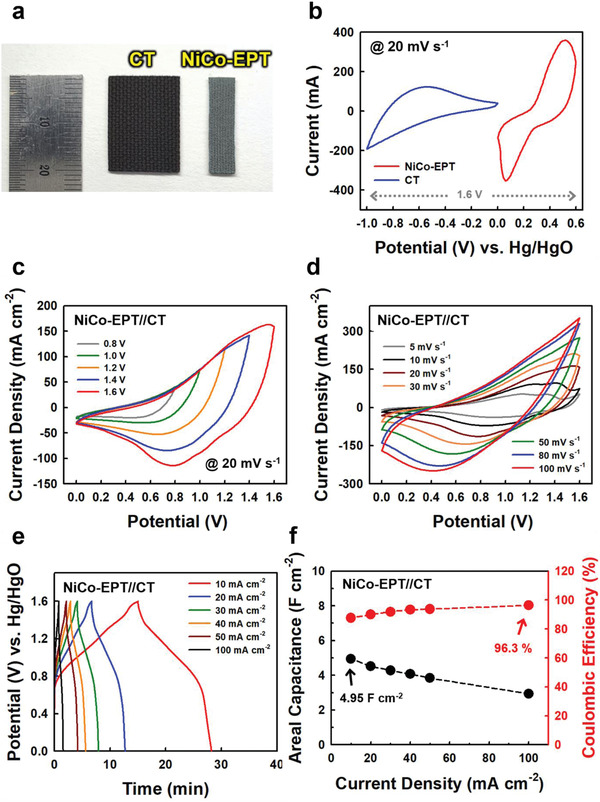
a) Digital images of NiCo‐EPT and CT for full‐cell fabrication considering the charge balance. b) CV curves of NiCo‐EPT and CT at a scan rate of 20 mV s^−1^. c) Potential window‐dependent CV curves of NiCo‐EPT//CT AFPs at a scan rate of 20 mV s^−1^. d) CV and e) GCD curves at various scan rates and current densities of NiCo‐EPT//CT AFPs. f) Areal capacitance and Coulombic efficiency of AFPs as a function of current density.

Based on these results, we tried to prepare asymmetric full‐cell pseudocapacitors (AFPs) composed of a 1‐stacked NiCo‐EPT cathode and the corresponding anode. To this end, a carbonized textile (CT) with a sheet resistance of approximately 6 Ω sq^−1^ and a highly porous structure was used as an anode to meet the electrochemical performance of the cathode (i.e., NiCo‐EPT) (Figure S30, Supporting Information). In this case, the CV curves of CT anode with broad oxidation peak at various scan rates indicated that the charge storage mechanism of CT anode originated from EDLC and reversible Faradaic reaction (by organic residues including hydroxyl groups or other oxygen groups).^[^ ^64^, ^65^, ^66^ ^]^ The areal capacitance of CT was measured to be approximately 2.4 F cm^−2^ at a current density of 10 mA cm^−2^ (Figure S31, Supporting Information). By considering the charge balance (q^+^ = q^−^) of each electrode, the area of the CT anode should be approximately 2.5 times larger than that of the NiCo‐EPT cathode to fabricate AFPs (i.e., NiCo‐EPT//CT) (**Figure** **5**a). In line with these charge balances and CV curves in the three‐electrode system, the NiCo‐EPT and CT showed stable electrochemical behavior in the potential ranges of 0 to 0.6 V and −1.0 to 0 V, respectively. Based on these results, the potential window of AFPs could be extended up to 1.6 V in KOH solution (Figure 5b). Additionally, the CV curves of AFPs measured from different operation voltage windows ranging from 0.8 to 1.6 V at a scan rate of 20 mV s^−1^ revealed a stable pseudocapacitive feature with reversible Faradaic reaction (Figure 5c). The CV curves of AFPs exhibited both EDLC and pseudocapacitive characteristics at various scan rates from 5 to 100 mV s^−1^ (Figure 5d). In this case, the CV curves of AFPs were gradually distorted with broad redox peaks with increasing the scan rate, indicating that the surface‐controlled charge storage mechanism dominates over the diffusion‐controlled process due to the limited ion diffusion kinetics at a faster sweep rate. Additionally, quasi‐triangular symmetric GCD profiles from 10 to 100 mA cm^−2^ indicated desirable capacitive features and excellent rate capabilities (Figure 5e). Particularly, the AFPs exhibited high areal (specific and volumetric) capacitances of approximately 4.95 F cm^−2^ (967 F g^−1^ and 49.5 F cm^−3^) and 2.94 F cm^−2^ (574 F g^−1^, 29.4 F cm^−3^) at current densities of 10 and 100 mA cm^−2^, respectively, and delivered an excellent Coulombic efficiency of 96.3% at a current density of 100 mA cm^−2^ (Figure 5f). Additionally, the areal energy densities of our devices were estimated to be approximately 1.76 mW h cm^−2^ at a power density of 8 mW cm^−2^ and 1.05 mW h cm^−2^ at 80 mW cm^−2^, respectively. These performance values were much higher than those of previously reported AFPs based on 3D substrates (Figure S32 and Table S2, Supporting Information). It should be noted that the AFPs with the abovementioned high areal capacitance and rate capability cannot be easily realized with other pseudocapacitive cathodes. Therefore, these results evidently demonstrated that NiCo‐EPT‐based AFPs can potentially be applied to various power applications with high performance.

## 3 Conclusion

We demonstrated that a high‐performance 3D fibril‐type textile pseudocapacitor with extremely high areal capacitance and good rate capability could be successfully prepared through a chalcogenide NP assembly‐induced electroplating process. For the preparation of conductive textiles, TOABr‐CuS NPs were assembled with NH_2_/SH‐functionalized Cys molecular ligands onto cotton textiles through a ligand exchange reaction, which minimized the separation distance (i.e., contact resistance) between adjacent CuS NPs. An additional Ni electroplating step converted conductive textiles to metallic textiles (≈0.06 Ω sq^−1^) that could be directly used as TCCs. Furthermore, the NiCo‐LDH layer was successively electroplated for the pseudocapacitor cathode, which still maintained the flexible/porous properties of pristine cotton textiles. Owing to their large surface area and bulk metal‐like conductivity, the formed NiCo‐EPT exhibited a considerably high areal capacitance of 12.2 F cm^−2^ at 10 mA cm^−2^ and high Coulombic efficiency. Additionally, this high areal capacitance could be further increased to 28.8 F cm^−2^ through a simple multi‐stacking method. These results evidently demonstrate that the NiCo‐EPTs prepared through our approach can possess high electrical conductivity and maintain a highly porous structure despite the high mass loading of pseudocapacitive components on TCCs (or multi‐stacked TCCs), resulting in high‐performance textile cathodes with unprecedently high areal capacitance and good rate capability. We believe that our approach can be easily applied to various high‐performance electrochemical electrodes including pseudocapacitor electrodes that require a large surface area, a high electrical conductivity, and facile charge transfer.

## Conflict of Interest

The authors declare no conflict of interest.

## Author Contributions

W.C. and D.N. contributed equally to this work. W.C., D.N., Y.K., and J.C. conceived the idea and designed the experiments. W.C. and D.N. performed all the experiments. C.H.K. performed the electrochemical measurements. W.C., D.N., Y.K., and J.C. wrote the manuscript. All authors discussed the results and commented on the manuscript.

## Supporting information

Supporting Information

## Data Availability

The data that support the findings of this study are available from the corresponding author upon reasonable request.
