# Supplementary material for: Fibril‐Type Textile Electrodes Enabling Extremely High Areal Capacity through Pseudocapacitive Electroplating onto Chalcogenide Nanoparticle‐Encapsulated Fibrils
Source: Adv Sci (Weinh). 2022 Sep 25;9(33):2203800. doi: 10.1002/advs.202203800 (PMC9685452; doi:10.1002/advs.202203800)
Supplement: Supplementary file 1 — Supporting Information [file ADVS-9-2203800-s001.pdf]

Supporting Information

Fibril-Type Textile Electrodes Enabling Extremely High Areal Capacity through Pseudocapacitive Electroplating onto Chalcogenide Nanoparticle-Encapsulated Fibrils

Woojae Chang,† Donghyeon Nam,† Seokmin Lee, Younji Ko, Cheong Hoon Kwon, Yongmin Ko, and Jinhan Cho**

Experimental Section

Materials: Sodium thiosulfate ($Na_2S_2O_3$, 99%), Nickel(II) chloride hexahydrate ($NiCl_2·6H_2O$), Palladium(II) chloride (PdCl2, 99%), Sodium hypophosphite monohydrate ($NaPO_2H_2·H_2O$, ≥99%), Ammonium chloride (NH_4Cl, ≥99.5%), Sodium citrate dihydrate ($Na_3C_6H_5O_7·2H_2O$, ≥99%), and Potassium hydroxide (KOH, 90%) were purchased from Sigma-Aldrich. Copper(II) chloride dihydrate ($CuCl_2·2H_2O$, ≥99%), Tetra-n-octylammonium bromide (TOABr, ≥98%). Nickel(II) sulfate hexahydrate ($NiSO_4·6H_2O$, ≥98%), Boric acid (H_3BO_3, ≥99.5%), Nickel(II) nitrate hexahydrate ($Ni(NO_3)_2·6H_2O$, 98%), Cobalt(II) nitrate hexahydrate ($Co(NO_3)_2·6H_2O$, 98.0−102.0%), and Tin(II) chloride dihydrate ($SnCl_2·2H_2O$, 98.0−103.0%) were purchased from Alfa Aesar. Additionally, Cysteamine (Cys, ≥95%), and Sodium Borohydride ($NaBH_4$, ≥98%) were received from TCI Co. All chemical reagents were used without further purification.

Synthesis of TOABr-CuS NPs: TOABr-CuS NPs in toluene were synthesized through a two-phase reaction. Briefly, 25 mM TOABr dispersed in Toluene (80 mL) and 33 mM $CuCl_2·2H_2O$ in deionized water (30 mL) were vigorously mixed for 15 min at 40 ℃.

Subsequently, 12 mmol $Na_2S_2O_3$ was added and stirred for 15 min which made the mixture solution to be clear. Then, 0.9 M $NaBH_4$, a reducing agent, in deionized water (10 mL) was poured in the solution. After 30 min, the deep brown toluene phase was separated from the mixture and washed several times with 10 mM HCl, 10 mM NH_4OH, and deionized water.

LbL assembly of (TOABr-CuS NP/Cys)$_n$ multilayers: Substrates including Si wafers, Au-sputtered Si wafers, quartz glasses, and QCM electrodes were irradiated with a UV ozone cleaner for 30 min. Above mentioned substrates and bare cotton textiles (0.5 x 3 cm^2) were first dipped into Cys solution (in ethanol, 2 mg mL^{-1}) for 30 min to form robust underlayer, then washed twice with pure ethanol to remove the weakly adsorbed Cys molecules. The formed Cys-coated substrates were dipped into TOABr-CuS NPs solution (in toluene, 30 mg mL^{-1}) for 30 min, then washed with pure toluene to eliminate the weakly adsorbed NPs. In the following process, TOABr-CuS NP coated substrates were dipped into Cys solution for 5 min to replace bulky TOABr ligands to small Cys ligands. After this ligand exchange reaction-induced adsorption process, weakly adsorbed Cys layer was re-washed out using pure ethanol. These processes were repeated until the desired bilayer number (n) of (TOABr-CuS NP/Cys)$_n$ multilayers was reached.

Preparation of Ni-EPT: Electroplating solution (Watts bath) was prepared using 240 g L^{-1} $NiSO_4{\cdot}6H_2O$, 45 g L^{-1} $NiCl_2{\cdot}6H_2O$, and 30 g L^{-1} H_3BO_3 with deionized water.[S1] (TOABr-CuS NP/Cys)$_5$-coated textiles (as a cathode) and nickel plate (as an anode) were immersed in the electroplating solution. Electroplating process was performed by adjusting the external current and electroplating time. The formed Ni-EPT was washed several times with deionized water, and then dried in a vacuum oven.

Preparation of Ni-CRT: Electroless deposition, using chemical reduction of Ni precursors on textile, was prepared from following process. 0.5 x 3 cm^2 sized cotton textile was first immersed in a sensitizing solution containing 50 mM $SnCl_2{\cdot}2H_2O$ and 0.15 M HCl (37 %) for 10 min. After this dipping process, the textile sample was dipped into an activating solution

containing 0.6 mM $PdCl_2$ and 0.03 M HCl. Finally, the sample was immersed in electroless plating bath held at 80 ℃, consisting of 240 g L^{-1} $NaH_2PO_2 \cdot H_2O$, 45 g L^{-1} $NiSO_4 \cdot 6H_2O$, 50 g L^{-1} NH_4Cl, 30 g L^{-1} $Na_3C_6H_5O_7 \cdot 2H_2O$, and adjusted pH to 9 with 0.27 % NH_4OH solution. After stirring for 120 min, sample was washed with deionized water and dried in a vacuum oven.

Preparation of NiCo-t: NiCo-LDH layer was additionally electroplated in a traditional 3-electrode electrochemical cell which is composed with Ni-EPT, Pt coil, and Ag/AgCl (3 M NaCl) as a working, counter, and reference electrode, respectively. Electroplating was carried out in a growth solution (10 mM $Ni(NO_3)_2 \cdot 6H_2O$ and 5 mM $Co(NO_3)_2 \cdot 6H_2O$ in deionized water) at a constant current density of 3 mA cm^{-2} for given electroplating time. After the process, the electrode was washed with deionized water, and then dried in a vacuum oven. In the case of performing the electroplating times for 15, 30 and 45 min, the formed electrodes were designated as NiCo-15, NiCo-30, and NiCo-45, respectively. Particularly, NiCo-60 is defined as NiCo-EPT because of the use of the same experimental condition (i.e., electroplating time ~60 min). In this case, the thickness of the NiCo-EPT was increased up to approximately 1.0 mm after electroplating process. For the stacked NiCo-EPTs, the upper part of each NiCo-EPT was attatched with silver paste until the desired stacking number was reached. After this process, the stacked NiCo-EPTs were fixed with epoxy.

Preparation of NiCo/Ni foam: Commercial Ni foam were ultrasonically cleaned in acetone, ethoanol, and deionized water for 30 min to remove organic residues on the surface, and then dried in vacuum oven for 24 h. After these cleaning processes, NiCo-LDH layer was electroplated onto the Ni foam using the the same electroplating conditions (3 mA cm^{-2} for 60 min) performed for the preparation of NiCo-EPT.

Preparation of L-NiCo-EPT: Ni was electroplated on $(TOABr\text{-}CuS\ NP/Cys)_5$-coated textiles using the external current density of 50 mA cm^{-2} for 10 min, which resultantly exhibited the relatively high sheet resistance of 20 Ω sq^{-1}. Subsequently, NiCo-LDH layer was

electroplated under the same condition as the preparation of NiCo-EPT (i.e., 3 mA cm^{-2} for 60 min).

Preparation of CT: Bare cotton textiles was heated at 100 °C for 30 min to remove residual moisture. In the following process, heating temperature was increased up to 950 °C at a rate of 3 °C min^{-1}, and then held for 3 hr. These processes were carried out in the furnace under a nitrogen atmosphere. After cooling to ambient temperature, the carbonized textile (CT) was taken out from the furnace. In this case, the formed CT exhibited a sheet resistance of 9 Ω sq^{-1}.

Preparation of the AFPs: NiCo-EPT and CT were composed of cathode and anode for AFPs, respectively. Area (or mass) ratio of cathode and anode was adjusted based on the following charge balance equation:[S2]

$$q = C \times \Delta E \times S \tag{1}$$

$$\frac{S^{+}}{S^{-}} = \frac{C^{-}\Delta E^{-}}{C^{+}\Delta E^{+}} \tag{2}$$

where q, C, ΔE, and S refers to stored charge, areal or specific capacitance (F cm^{-2} or F g^{-1}), potential window (V) during the CV or GCD operation, and area (cm^{2}) or mass (g) of the active material, respectively. Based on the equation, areal ratio of the NiCo-EPT and CT was calculated to be 2.54 : 1.

Characterization: The crystal structure and size distribution of TOABr-CuS NPs was investigated by HR-TEM (Tecnai 20, FEI). Surface morphology and EDX mapping were observed using FE-SEM (Quanta 250 FEG, FEI). The UV-vis absorbance spectra of multilayers on quartz glasses were recorded using Lambda 365 (Perkin Elmer) within a wavelength ranging from 200 to 1000 nm. The mass change (Δm, μg cm^{-2}) of each layer was quantitively acquired from the frequency change (ΔF, Hz) from QCM (QCM 200, SRS) using the Sauerbrery equation (3) as follows:[S3]

$$\Delta F\ (Hz) = -\frac{2F_0^2}{A\sqrt{\rho_q \mu_q}} \times \Delta M \qquad (3)$$

Where F_0 indicates the resonant frequency of fundamental mode of the crystal (5 MHz). A, ρ_q, and μ_q refers to electrode surface area (cm^2), density (2.65 g cm^{-3}), and shear modulus (2.95 x 10^{11} g cm^{-1} s^{-2}) of QCM electrode respectively. By considering each values, above equation can be simplified as equation (4):

$$-\Delta F = 56.6 \times \Delta M \qquad (4)$$

FTIR analysis of multilayers on Au-sputtered Si wafers was conducted using a Cary 600 (Agilent Technologies) operated in Advanced Grazing Angle (AGA) mode with at a 4 cm^{-1} resolution, and obtained data were plotted using spectrum analysis software (OMNIC, Thermo Fisher Scientific). X-ray diffraction (XRD) patterns were obtained using a SmartLab instrument (Rigaku) with Cu Kα radiation. X-ray photoelectron spectroscopy (XPS) analysis were performed by X-TOOL (ULVAC-PHI) with monochromatic Al Kα radiation as the excitation source. The sheet resistance was measured using four-probe measurement method with Loresta-GP MCP-T610 (Mitsubishi Chemical Analytech).

Electrochemical measurements: All the electrochemical measurements were carried out with an Ivium-n-Stat electrochemical workstation (Ivium Technologies). Electrochemical performances of all single electrodes were evaluated in a three-electrode cell system and asymmetric supercapacitor (ASCs) was evaluated in two-electrode cell system. In the three-electrode configuration, all single electrodes (active area ~0.5 x 1.5 cm^2), Pt mesh, and Hg/HgO (1M NaOH) were used as the working, counter, reference electrode, respectively. 6 M KOH was used as an electrolyte for three and two-electrode cell system. EIS measurements were performed in the frequency range of 10^5 to 0.1 Hz with a perturbation amplitude of 0.01 V.

The capacitance (C) values of the electrodes were calculated from the GCD discharge profiles according to the following equation (5):[S4]

$$C = \frac{I\Delta t}{S\Delta V} \tag{5}$$

Where I, Δt, and ΔV refers to applied current (A), discharging time (s), and operating potential window (V), respectively. The variable S indicates the area (cm^2), mass (g), or volume (cm^3) of the active material.

Warburg impedance coefficient (σ_w) was obtained from the slope values in the following equation (6): [S5]

$$Z' = R_s + R_{ct} + \sigma_w \omega^{-0.5} \tag{6}$$

Where ω indicate angular frequency.

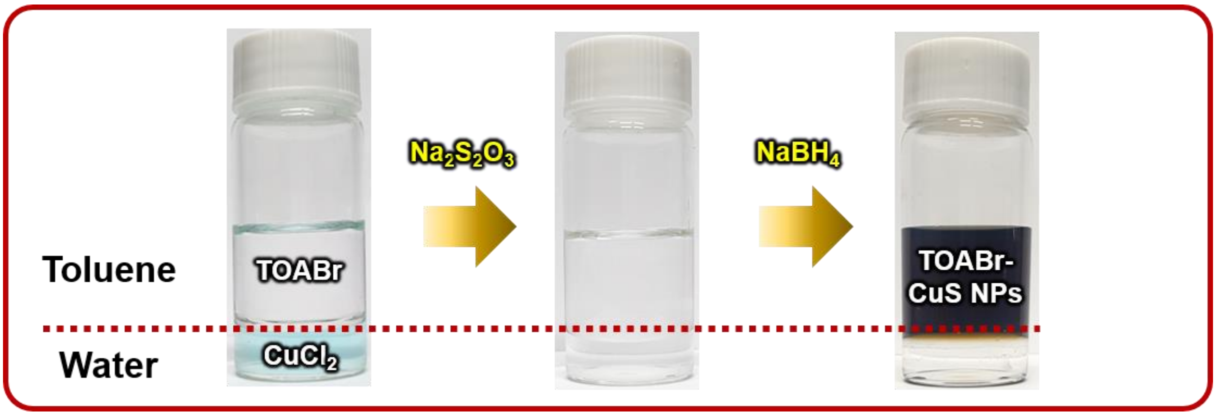

Figure S1. Photographic images of TOABr-CuS NPs synthetic process, which showed stable dispersion in toluene without particle aggregations.

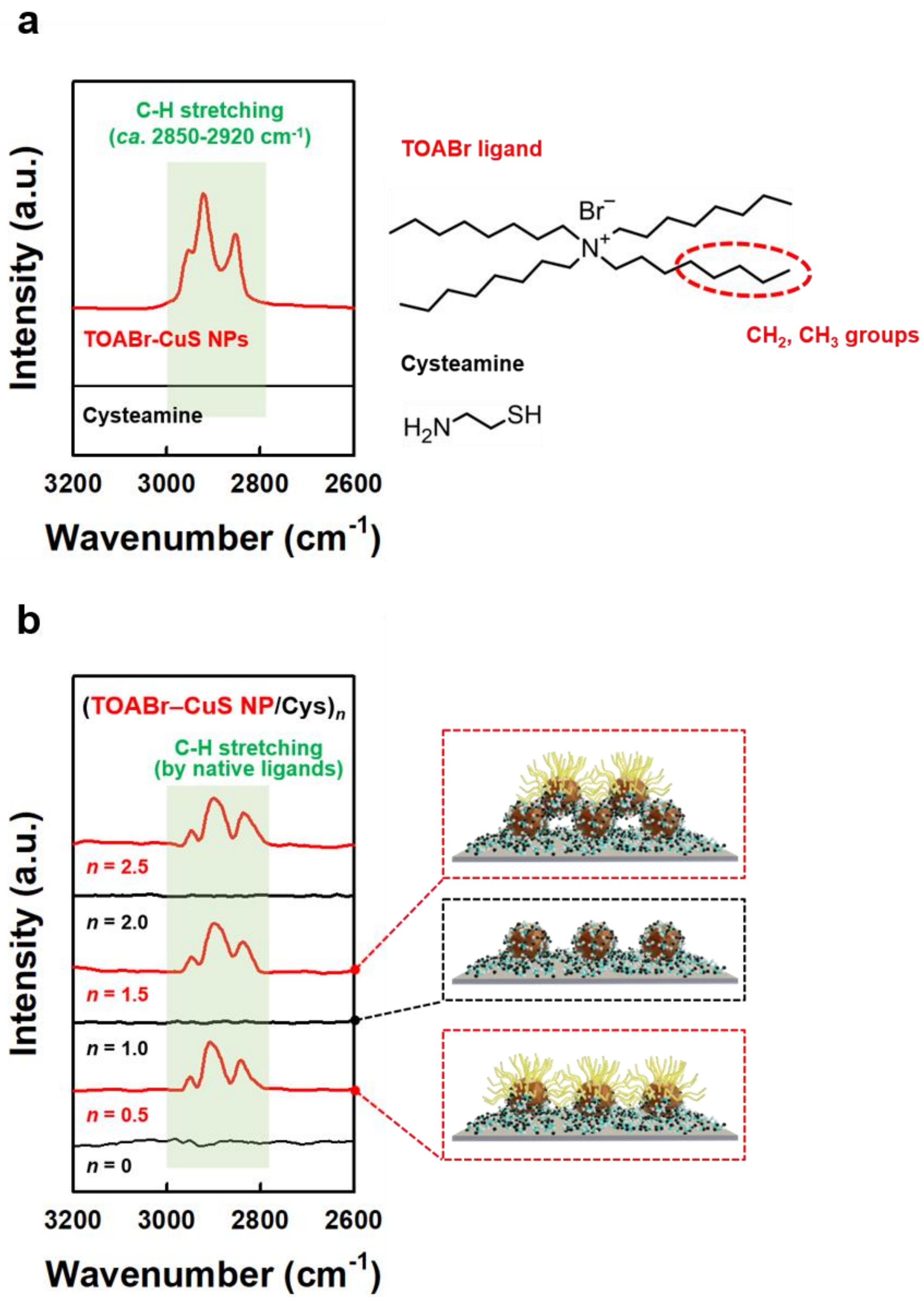

Figure S2. FT-IR spectra and schematic illustration of a) pristine TOABr-CuS NPs, cysteamine solution and b) LbL-assembled (TOABr-CuS NP/Cys)$_n$ multilayers as a function of the bilayer number (n).

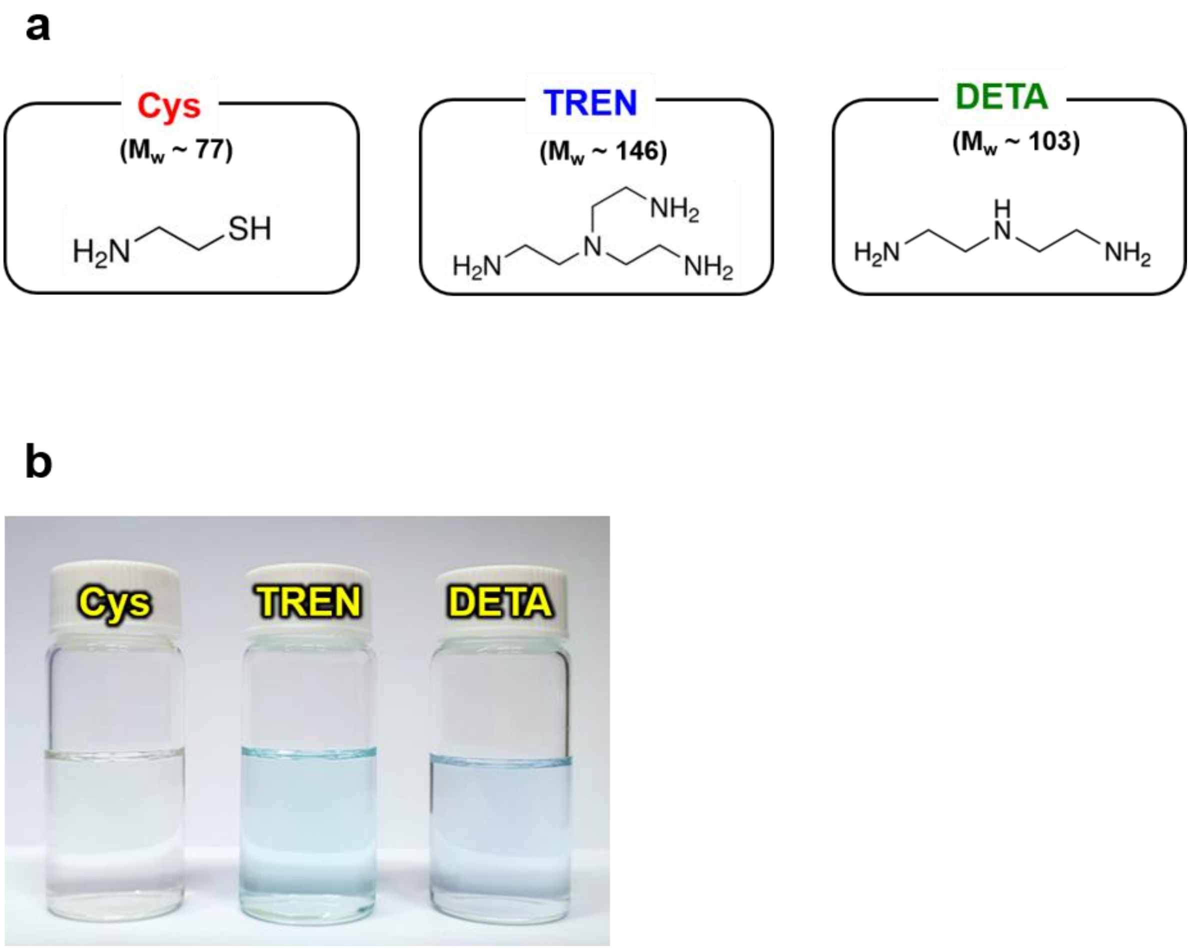

Figure S3. a) Molecular structures and weight (M_w) of Cys, TREN, and DETA as a small molecular ligand for TOABr-CuS NPs-based multilayers. b) Photographic images of Cys, TREN, DETA solution after dipping TOABr-CuS NPs-deposited wafers. After dipping, Cys solution remained transparent, but the colors of TREN and DETA solution turned blue, implying that Cu ions have been disassociated from CuS NPs.

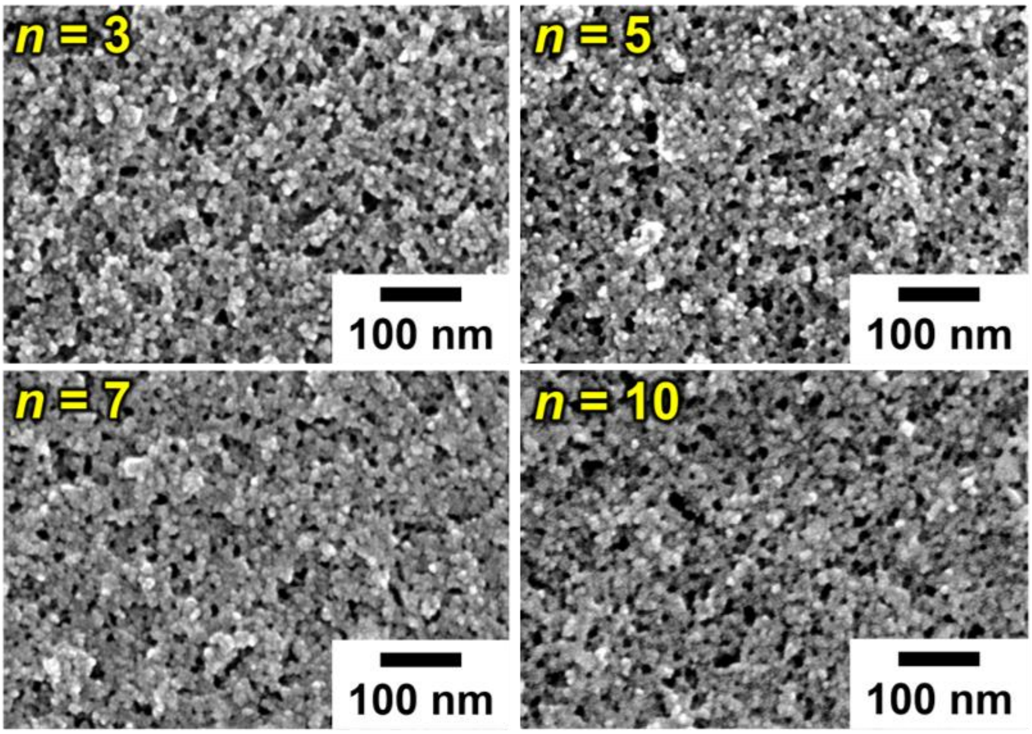

Figure S4. Planar FE-SEM images of (TOABr-CuS NP/Cys)$_n$ multilayers as a function of the bilayer number (n = 3, 5, 7, 10).

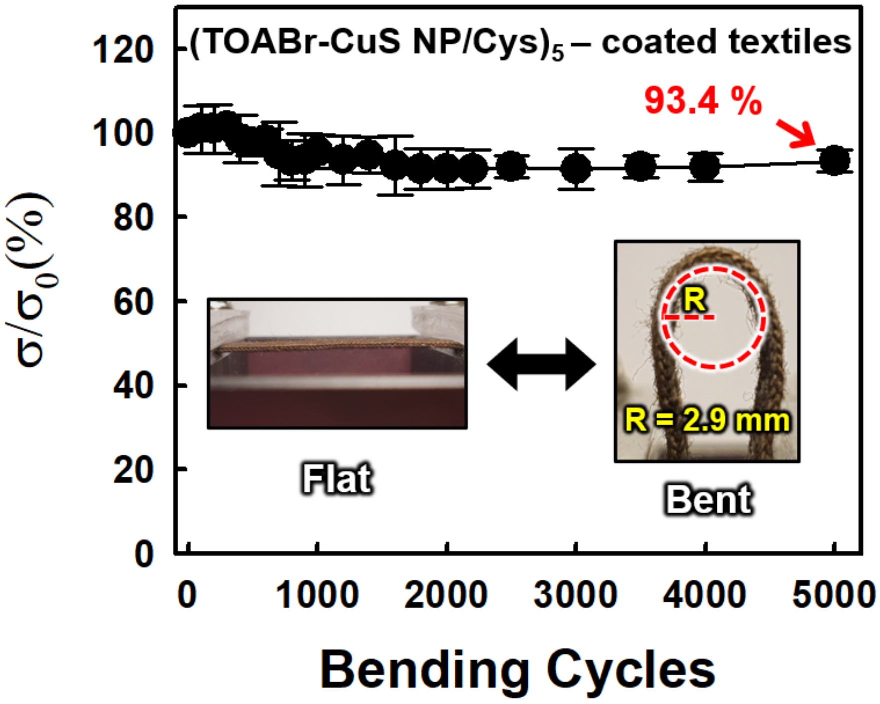

Figure S5. Relative electrical conductivity (σ/σ_0) of (TOABr-CuS NP/Cys)$_5$-coated textiles as a function of the bending cycles (bending radius of ~2.9 mm).

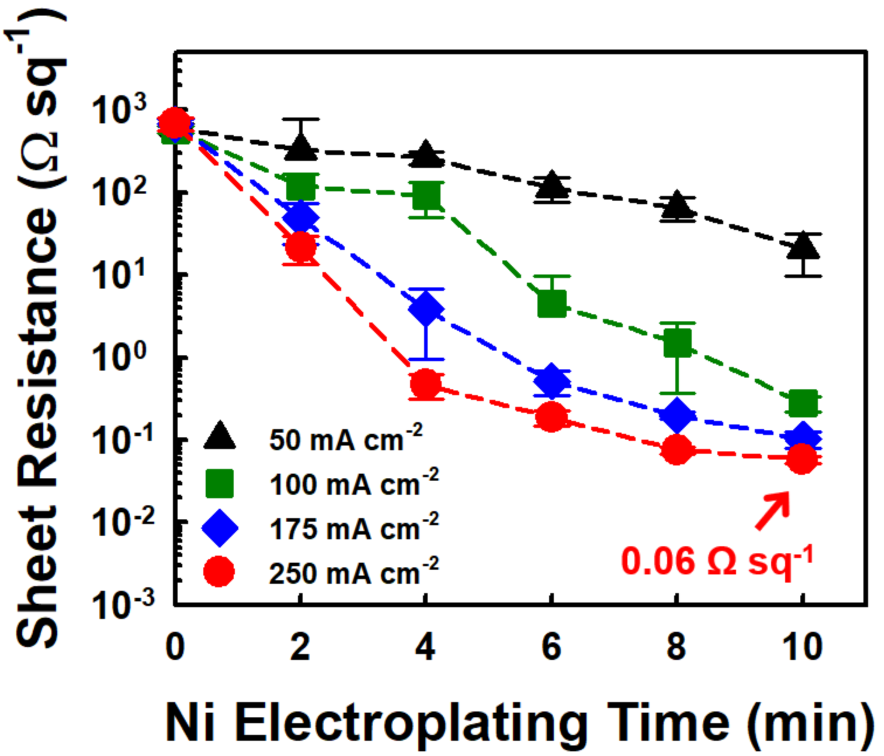

Figure S6. Sheet resistance of Ni-electroplated (TOABr-CuS NP/Cys)$_5$-coated textiles as a function of external current density and electroplating time.

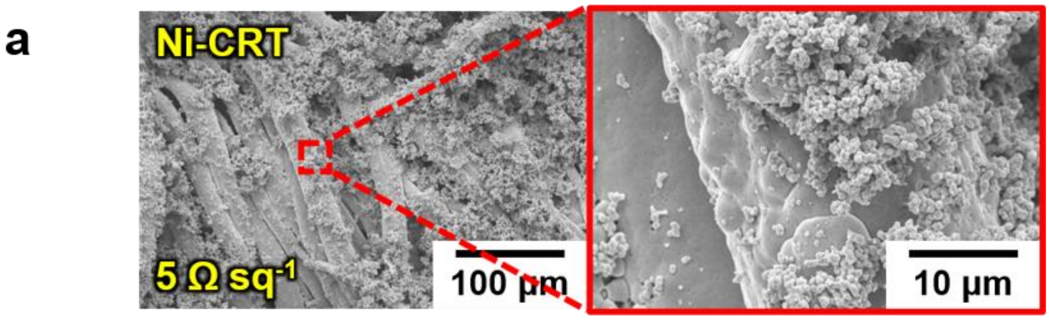

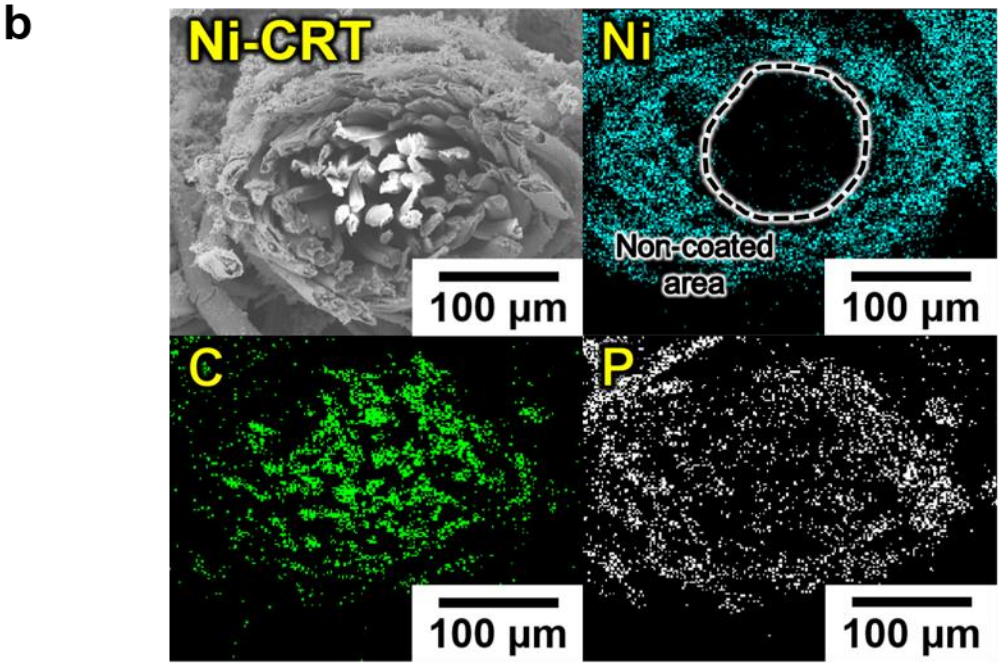

Figure S7. a) Planar FE-SEM and b) cross-sectional EDX mapping images of Ni-CRT. When Ni ions adsorbed onto the hydroxyl group (-OH) functionalized cotton textiles, conductive seeds are sparsely formed due to the electrostatic repulsion between the Ni ions with the same charge. Subsequent Ni ions continuously incorporated into this conductive seed for nucleation growth, resulting non-uniform Ni coating from the exterior to the interior of the textiles.

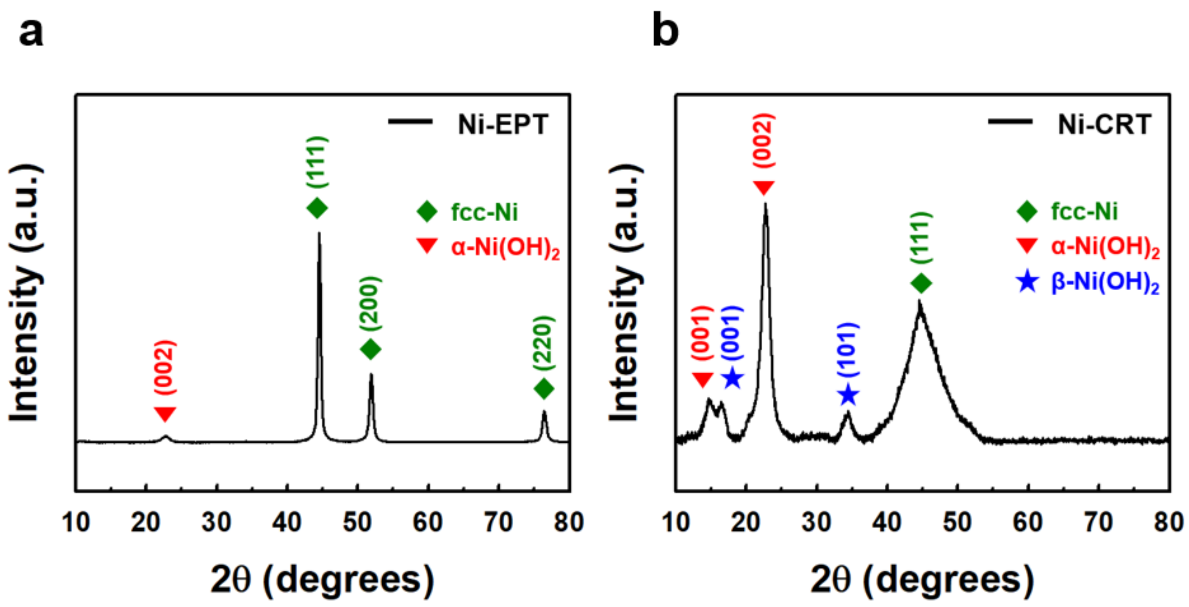

Figure S8. XRD spectra of a) Ni-EPT and b) Ni-CRT. In contrast to Ni-EPT, Ni-CRT clearly showed the presence of α, β-$Ni(OH)_2$, which attributed from partial surface oxidation. Especially, metastable phase of α-$Ni(OH)_2$ was dominantly formed than thermodynamically stable phase of β-$Ni(OH)_2$.

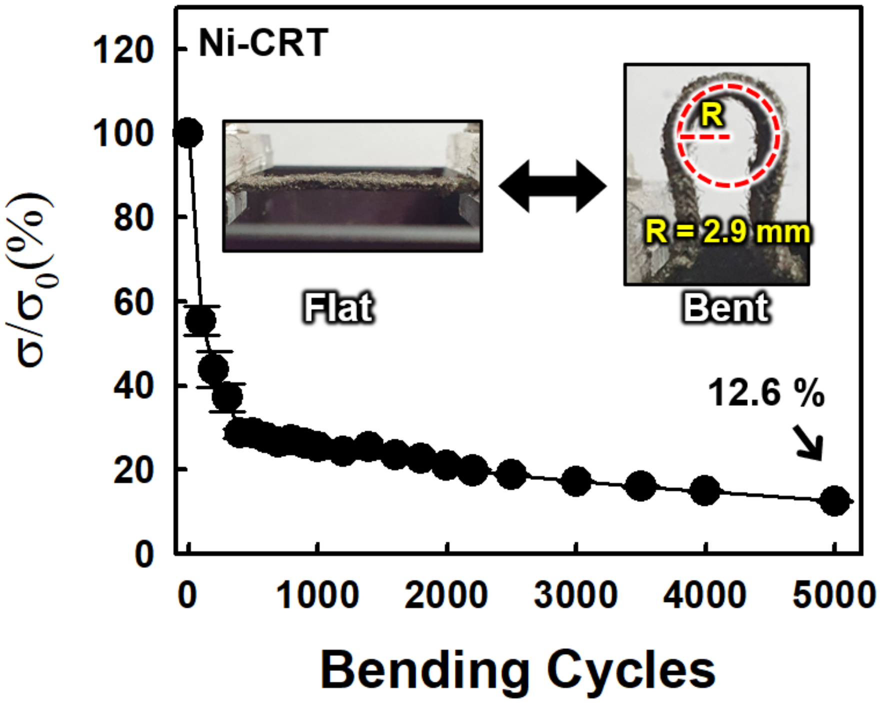

Figure S9. Relative electrical conductivity (σ/σ_0) of Ni-CRT as a function of the bending cycles (bending radius of ~2.9 mm). After 5000 bending cycles, Ni-CRT maintained 12.6 % of their initial electrical conductivity indicating low mechanical stability compared to the Ni-EPT.

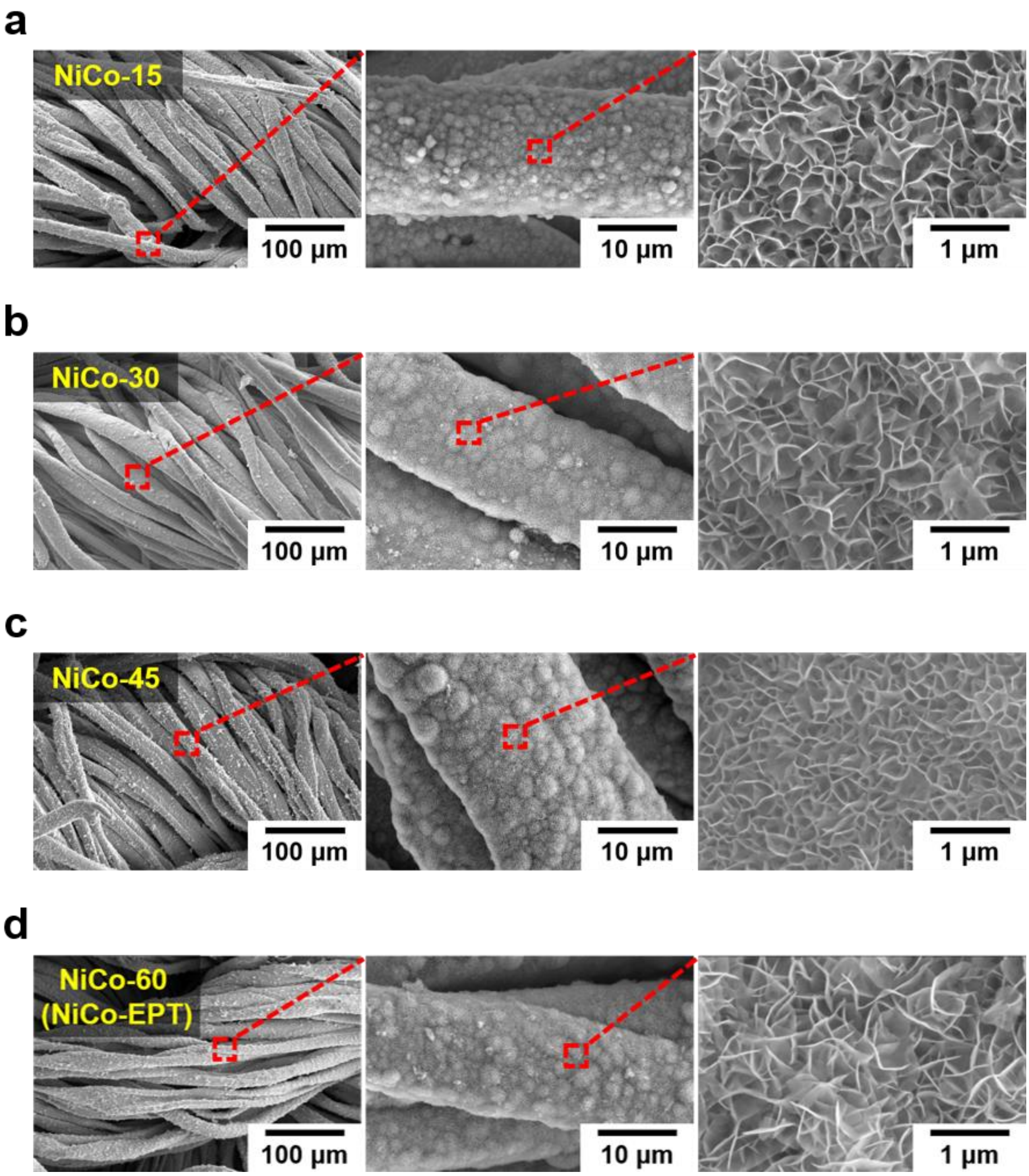

Figure S10. Planar FE-SEM images of a) NiCo-15, b) NiCo-30, c) NiCo-45, and d) NiCo-60 (NiCo-EPT).

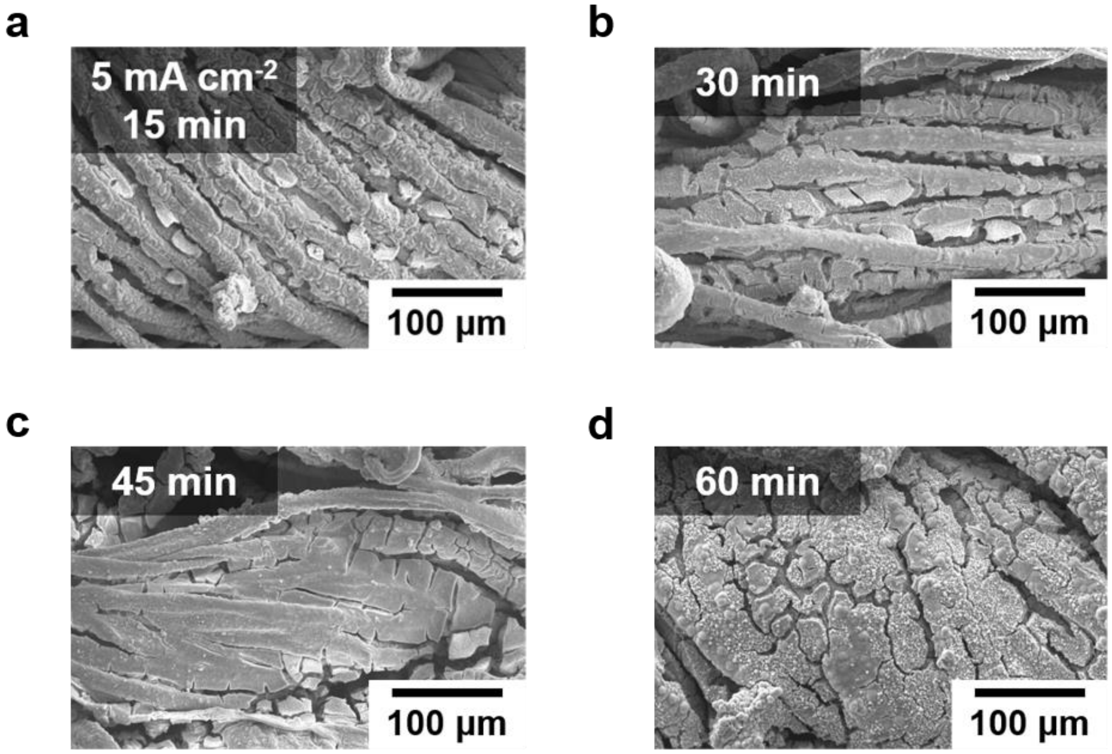

Figure S11. Planar FE-SEM images of time-dependent NiCo-LDH electroplating on Ni-EPT at a constant current density of 5 mA cm^{-2} for a) 15, b) 30, c) 45, and d) 60 min, respectively.

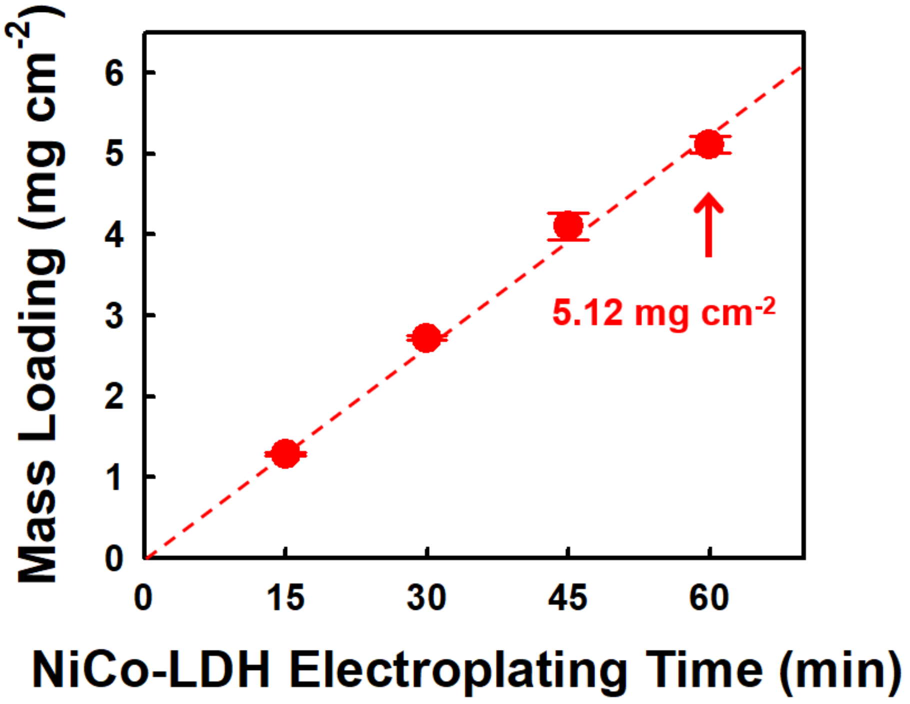

Figure S12. Mass loading of NiCo-LDH electroplated textiles as a function of electroplating time at 3 mA cm^{-2} (i.e., NiCo-t). For each NiCo-LDH electroplating time, they are designated as NiCo-15, NiCo-30, and NiCo-45, and NiCo-60 is the same condition as NiCo-EPT.

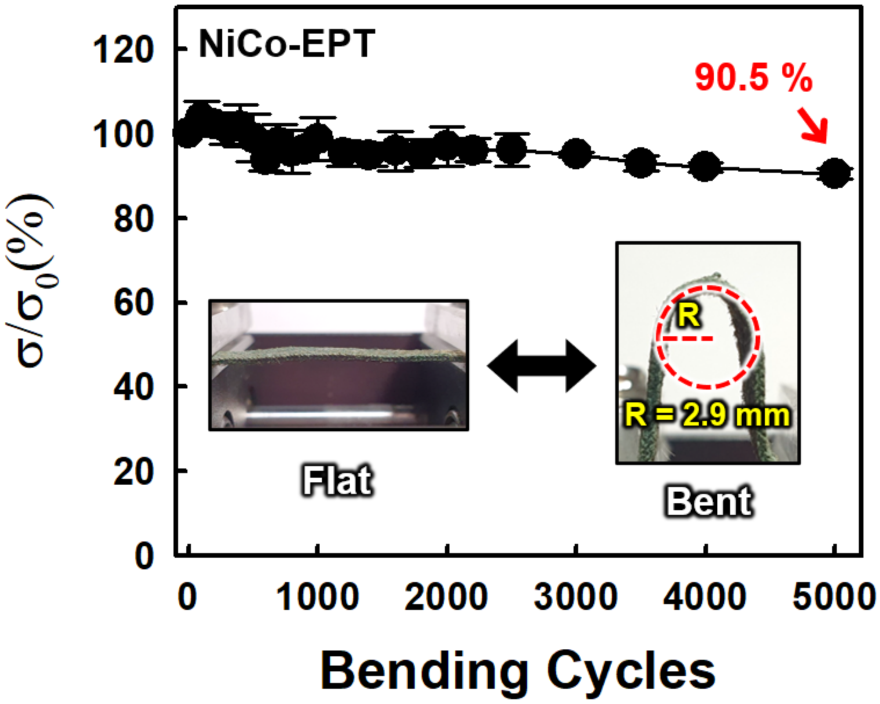

Figure S13. Relative electrical conductivity (σ/σ_0) of NiCo-EPT electrodes as a function of the bending cycles (bending radius of ~2.9 mm).

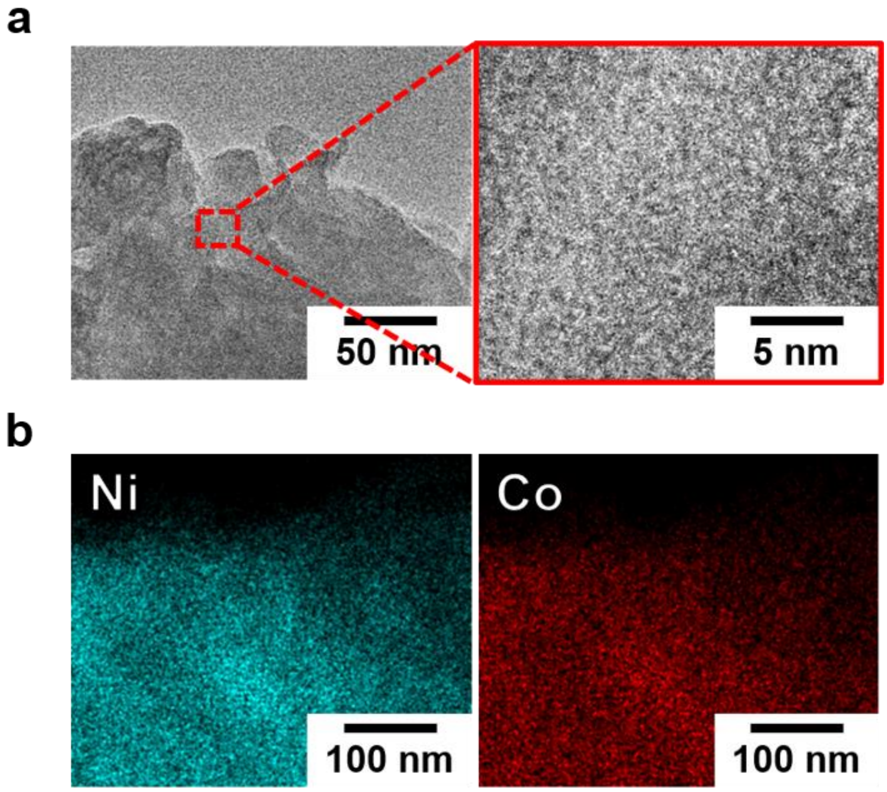

Figure S14. a) HR-TEM and b) EDX mapping images of NiCo-LDH layer.

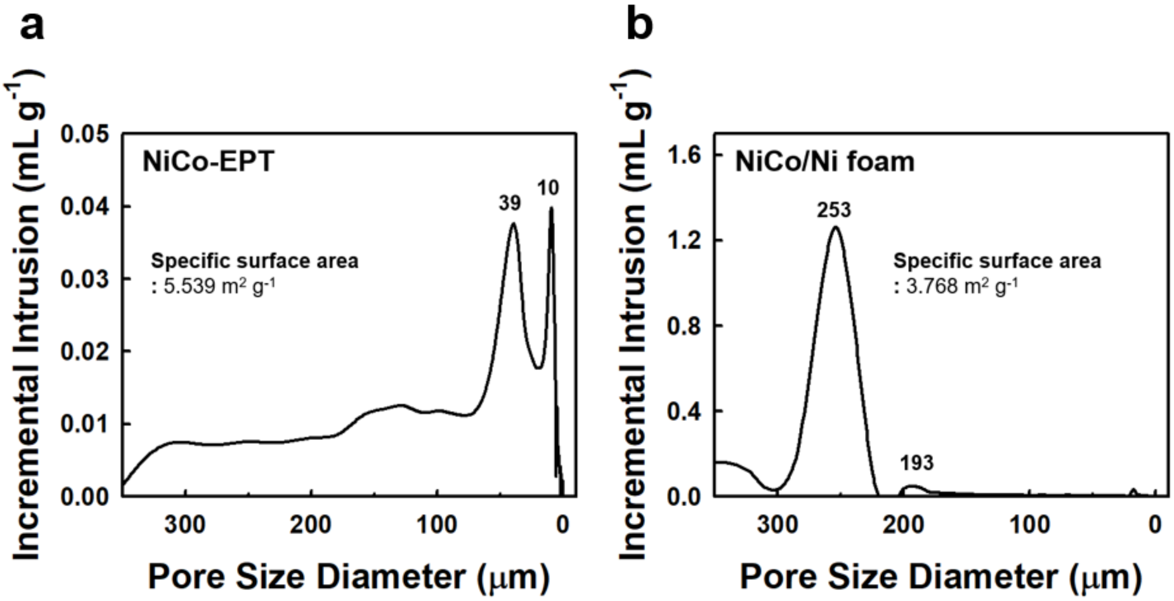

Figure S15. Incremental intrusion data of mercury vs. pore diameter of a) NiCo-EPT and b) NiCo/Ni foam investigated by mercury porosimetry technique. The pore size of NiCo-EPT exhibited distinct peaks at 10 and 39 µm, while NiCo/Ni foam had distinct peaks at 193 and 253 µm.

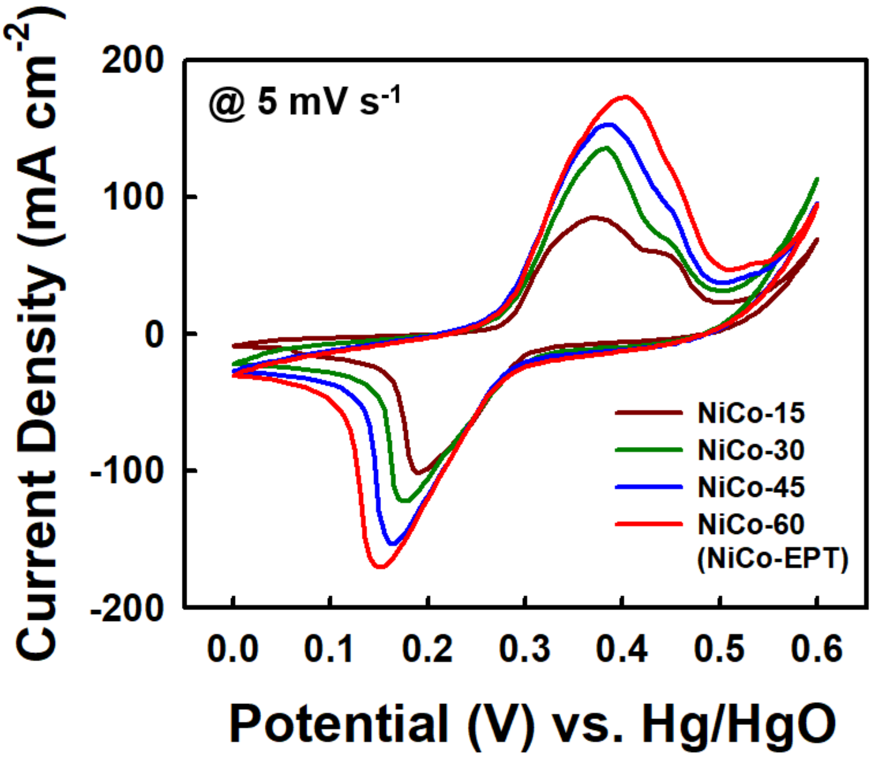

Figure S16. CV curves of NiCo-t electrodes at a scan rate of 5 mV s^{-1}.

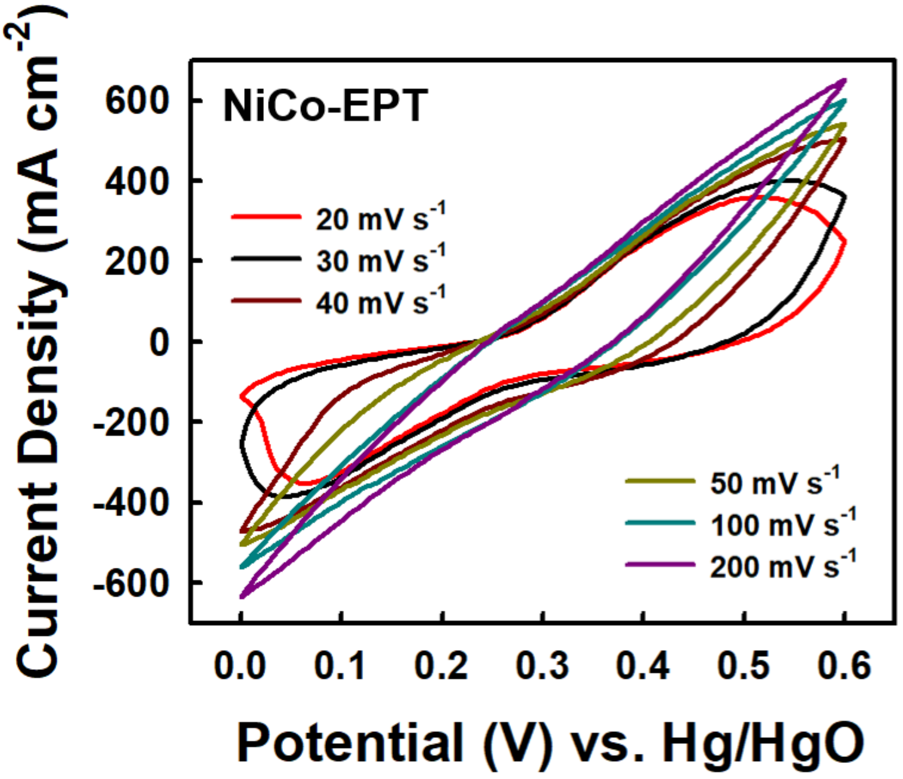

Figure S17. CV curves of NiCo-EPT electrodes at a various scan rates in the range from 20 to 200 mV s^{-1}

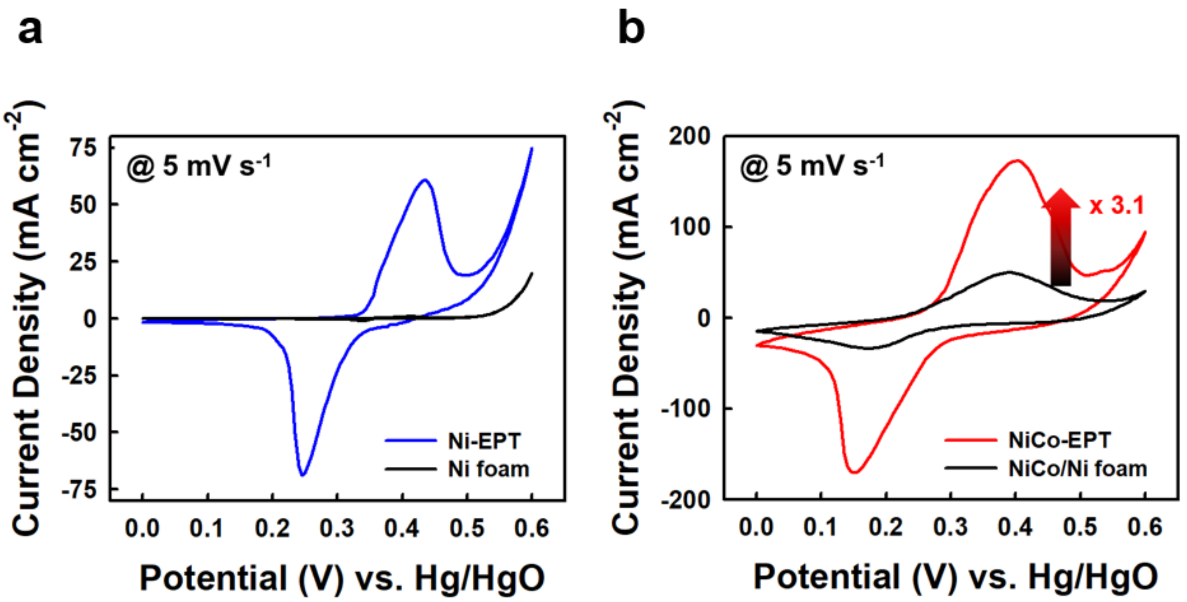

Figure S18. a) CV curves of Ni-EPT and Ni foam at a scan rate of 5 mV s^{-1}. b) CV curves of NiCo-EPT and NiCo/Ni foam electrodes at a scan rate of 5 mV s^{-1}.

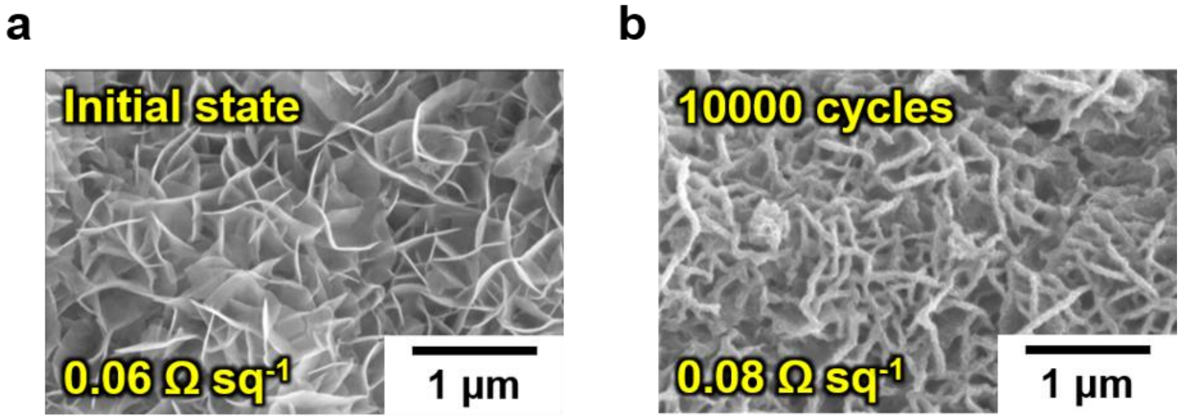

Figure S19. Planar FE-SEM images of NiCo-EPT at a) initial state and b) after 10,000 cycles of electrochemical stability test.

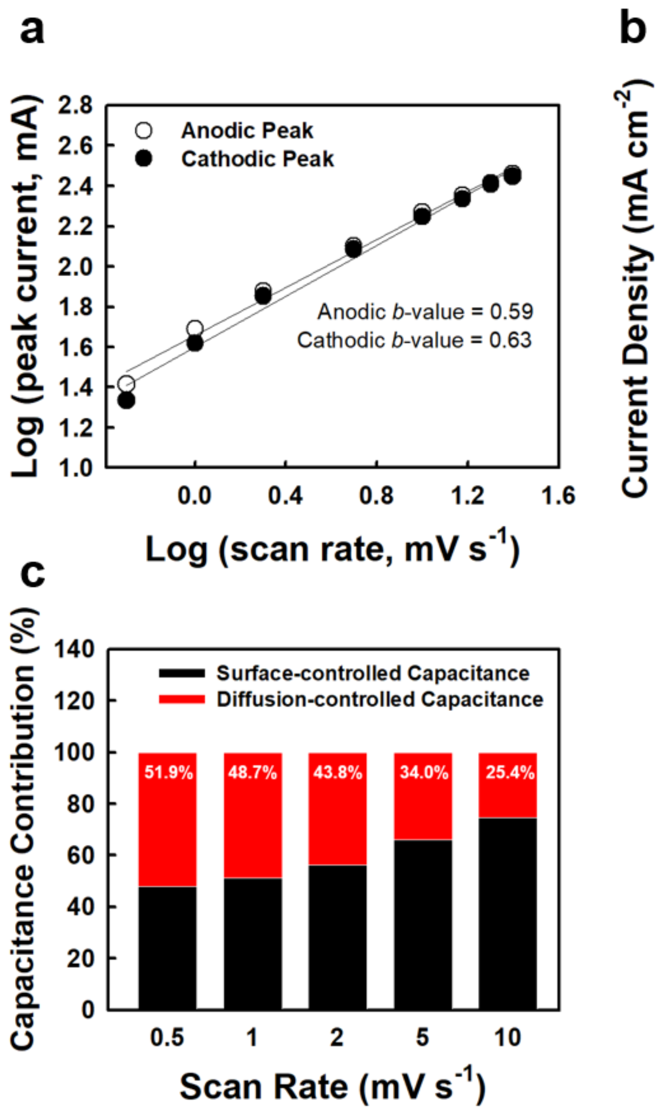

Figure S20 a) *log*(current, *i* (mA))–*log*(scan rate, *v* (mV s^{-1})) plots of the NiCo-EPT electrodes at anodic and cathodic peak currents. In this case, the slope represented the *b*-value. b) CVs of the NiCo-EPT electrode with the contribution of the surface-controlled charge reaction. c) Capacitance contribution of the NiCo-EPT electrode as a function of the scan rate ranging from 0.5 to 10 mV s^{-1}.

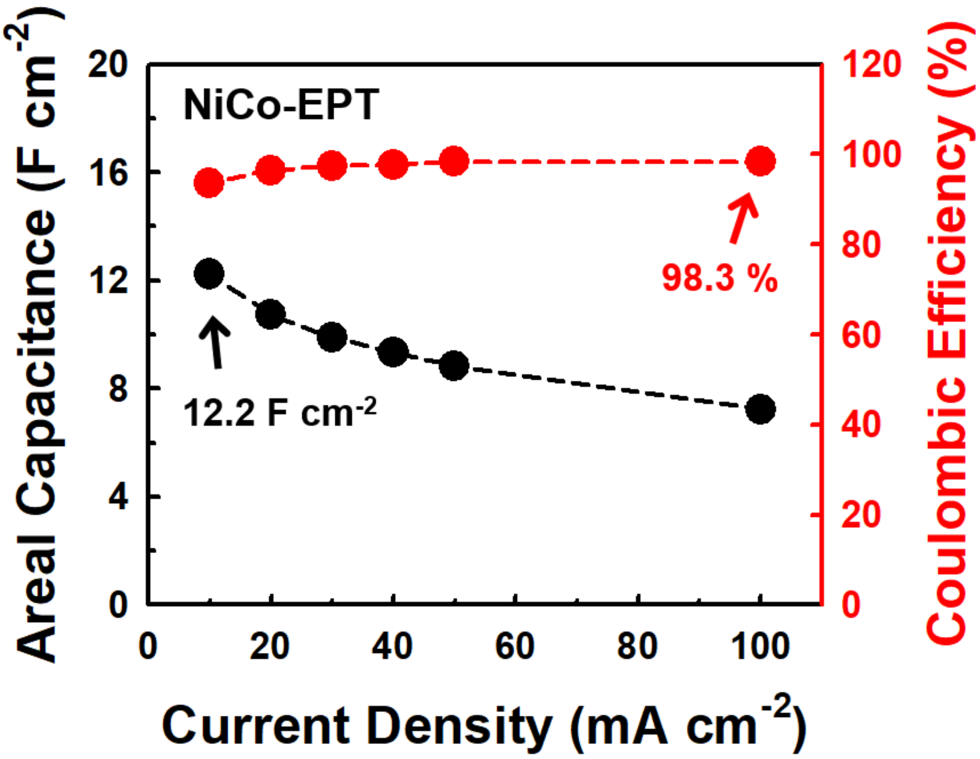

Figure S21. Areal capacitance and Coulombic efficiency values of the NiCo-EPT electrodes at a various current densities in the range of 10 to 100 mA cm^{-2}.

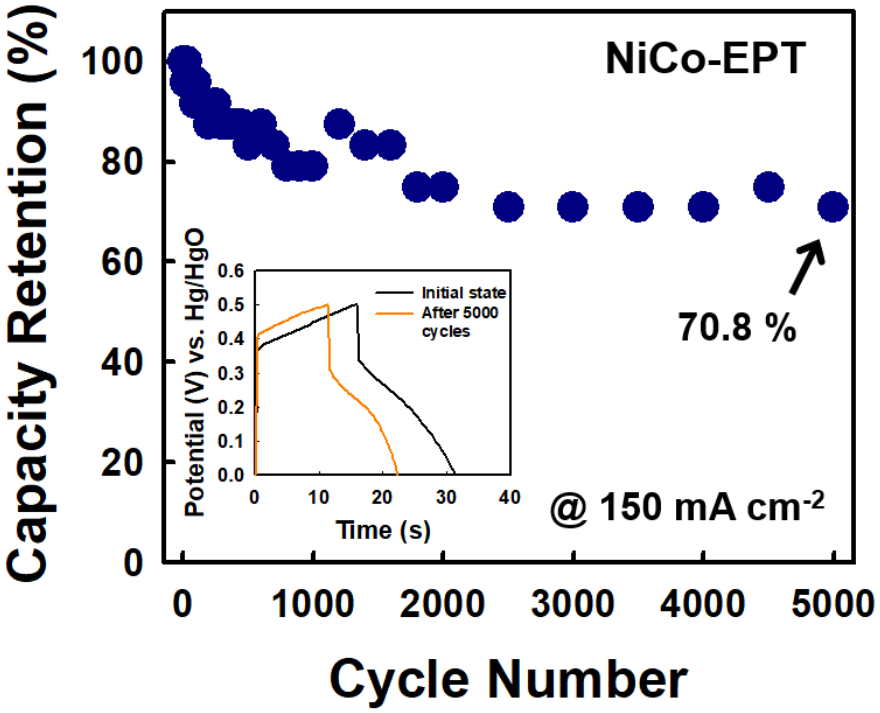

Figure S22 Capacity retention of NiCo-EPT electrodes at a current density of 150 mA cm^{-2}.

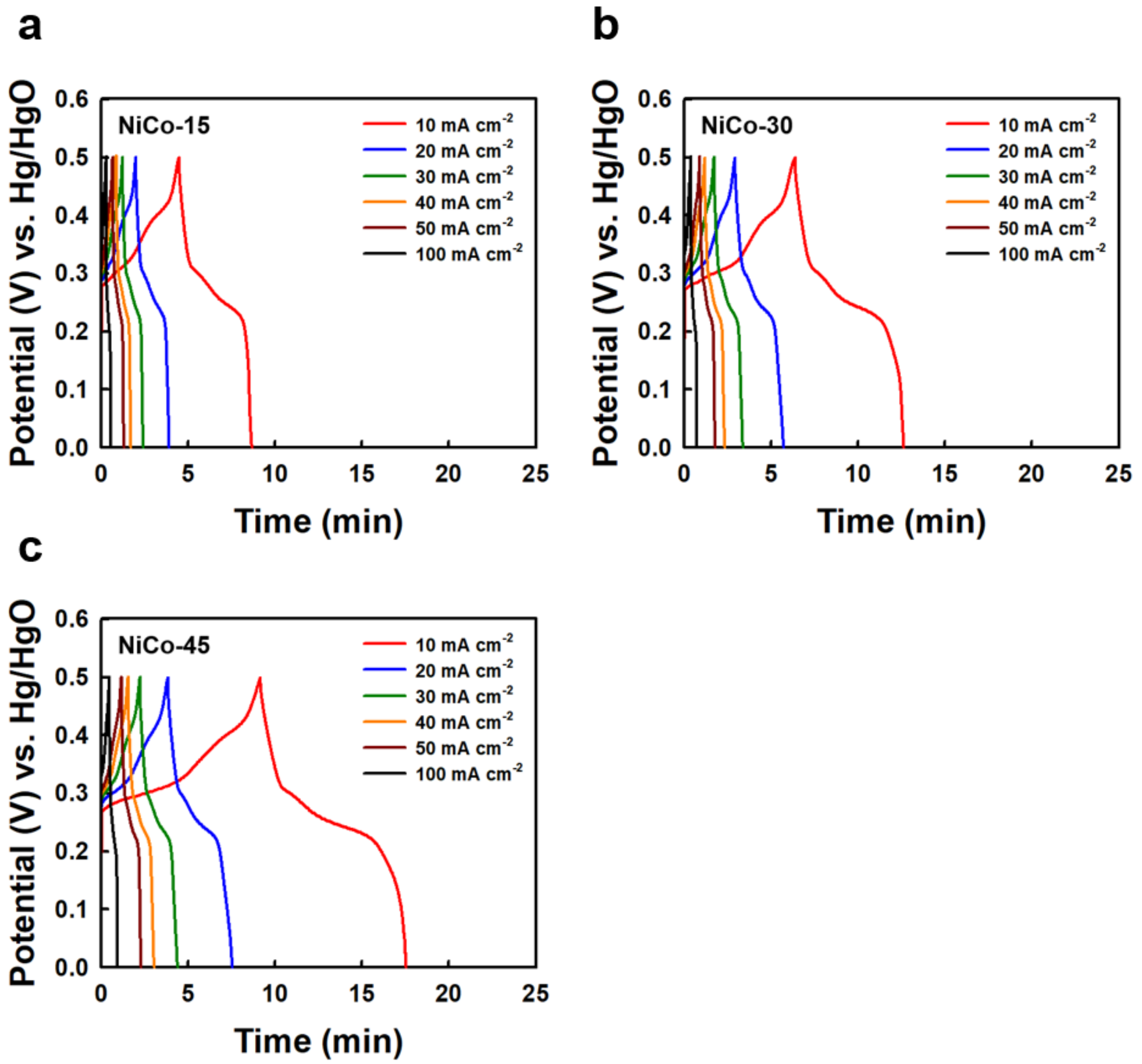

Figure S23. GCD curves of NiCo-t electrodes at a various current densities in the range of 10 to 100 mA cm^{-2}. a) NiCo-15, b) NiCo-30, c) NiCo-45 respectively.

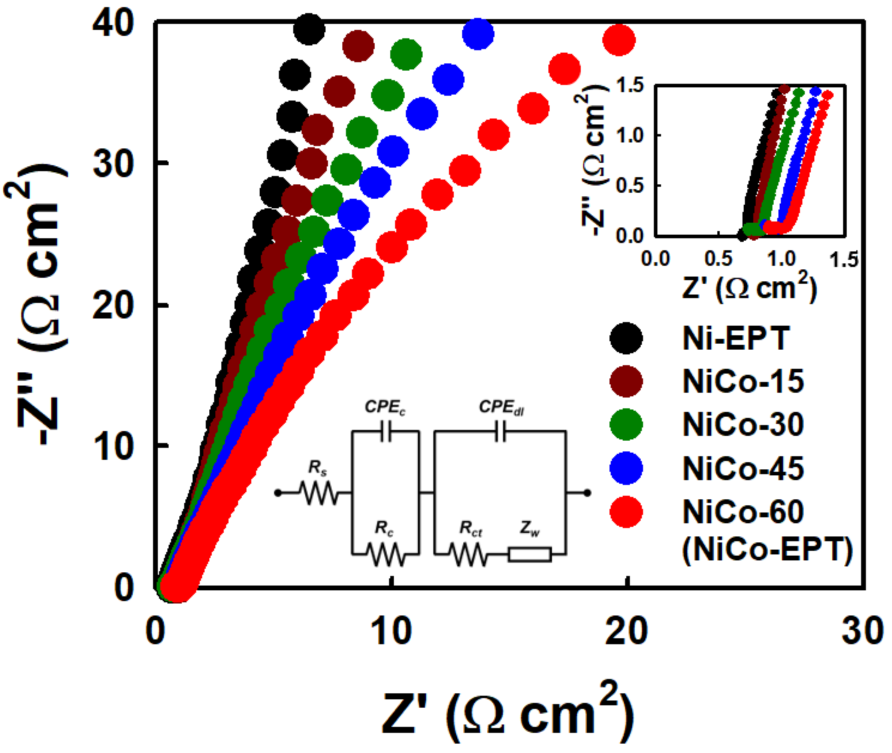

Figure S24. Nyquist plots of NiCo-t and Ni-EPT electrodes in the frequency range from 10^5 to 0.1 Hz, measured at 0.1 V (amplitude potential ~5 mV). The inset showed the Nyquist plots in the high frequency region and corresponding equivalent circuit model.

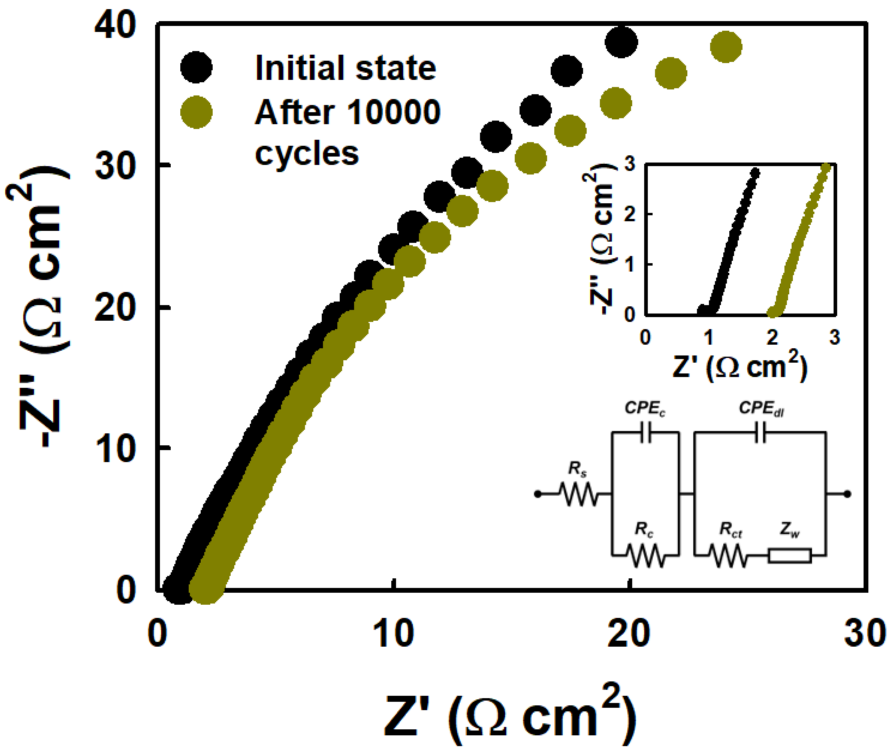

Figure S25. Nyquist plots of NiCo-EPT at initial state and after 10,000 cycles of electrochemical stability test in the frequency range from 10^5 to 0.1 Hz, measured at 0.1 V (amplitude potential ~5 mV). The inset showed the Nyquist plots in the high frequency region and corresponding equivalent circuit model.

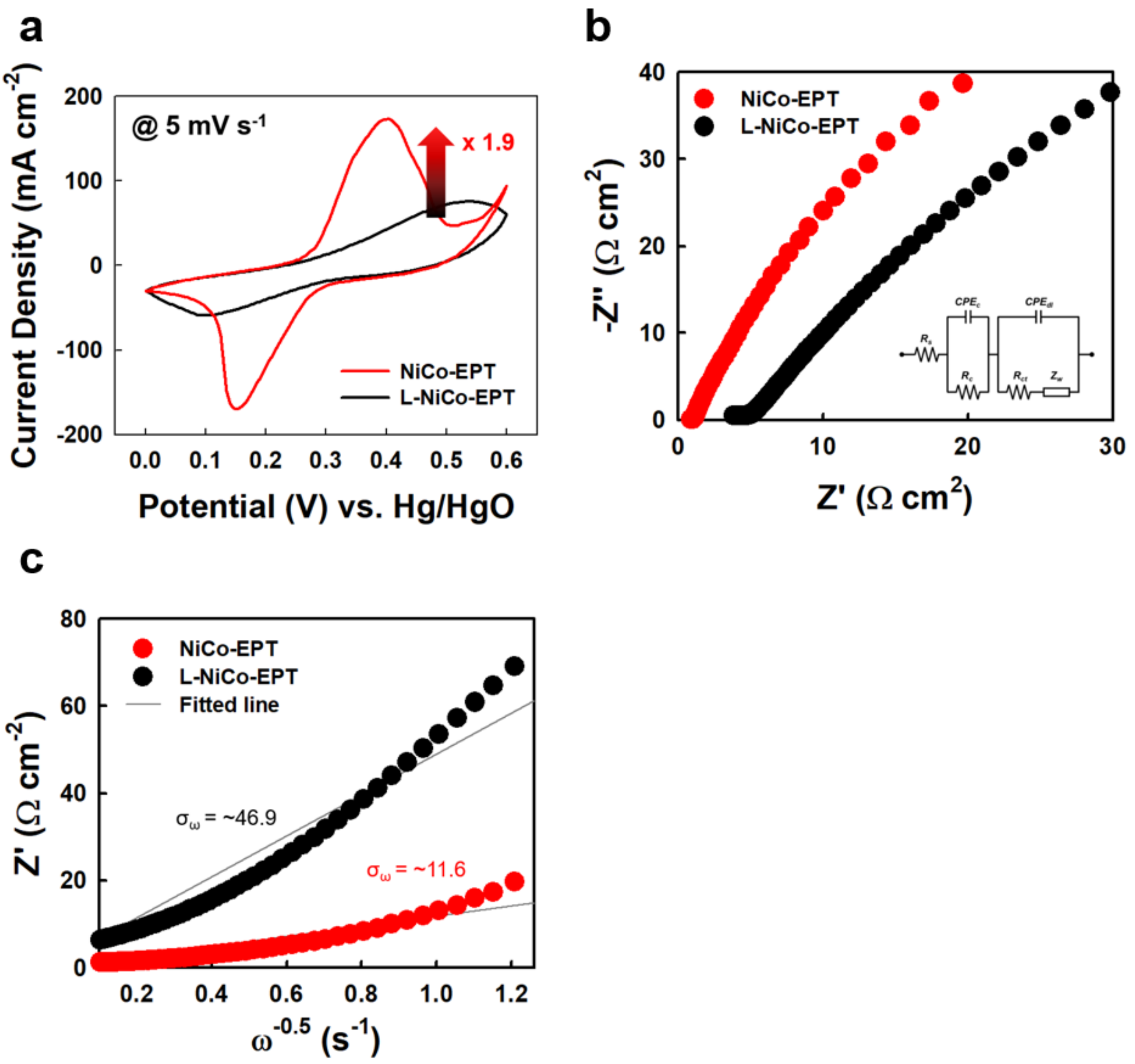

Figure S26. a) CV curves, b) Nyquist plots, and c) Warburg impedance coefficient plots of L-NiCo-EPT and NiCo-EPT.

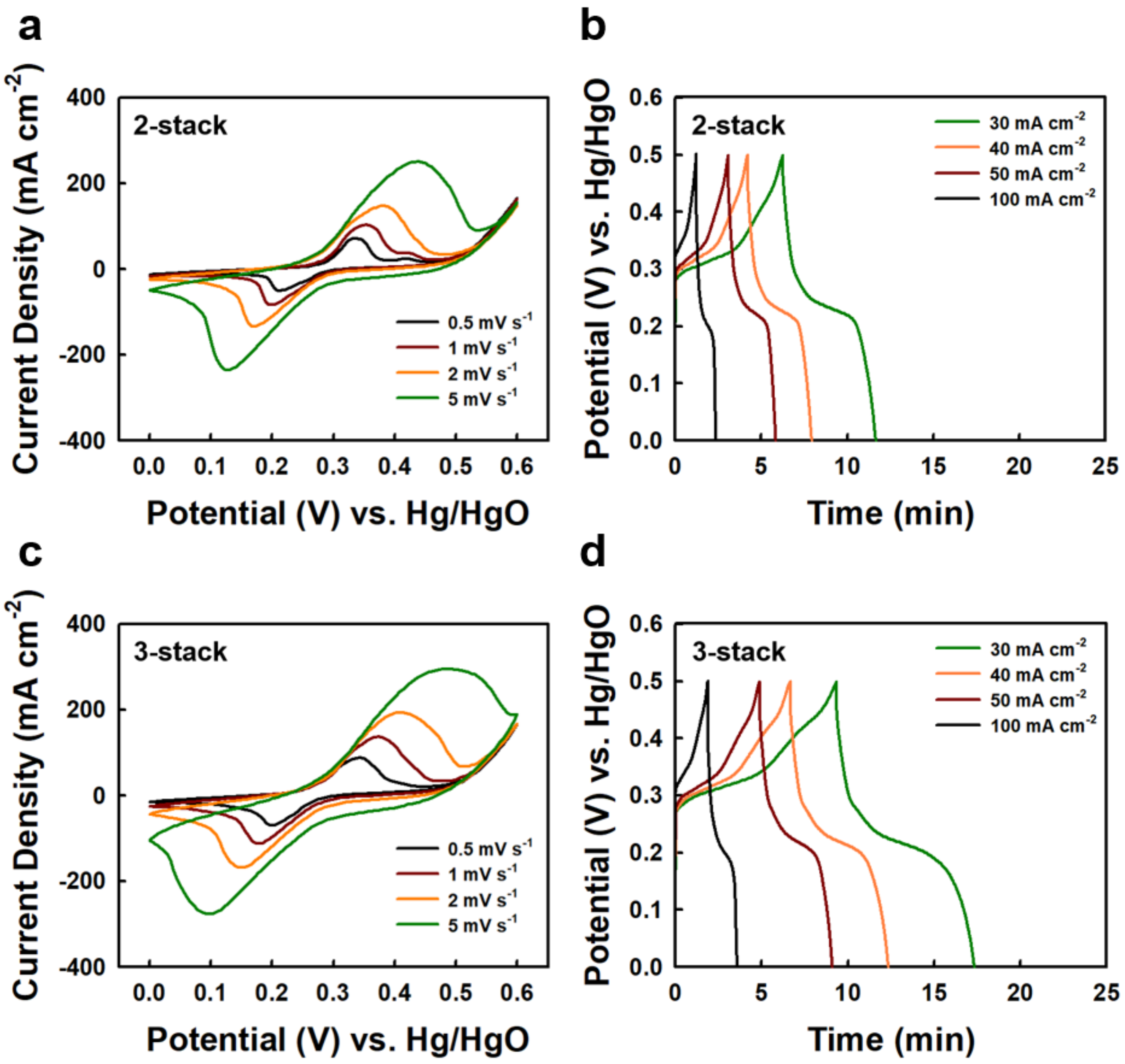

Figure S27. CV and GCD curves of multi-stacked NiCo-EPT with different stacking number. a), b) 2-stack and c), d) 3-stack, respectively.

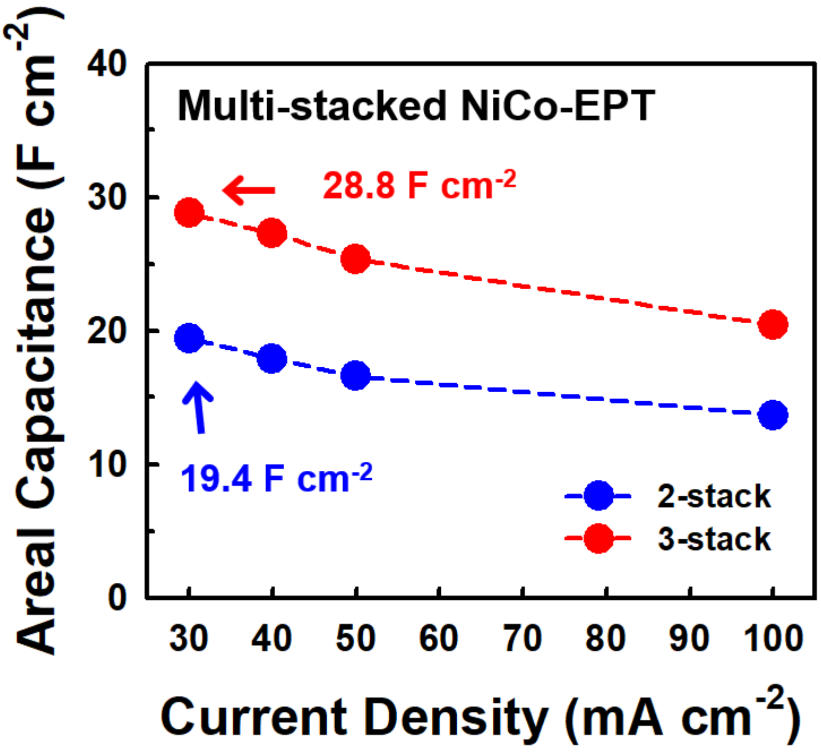

Figure S28. Areal capacitance of multi-stacked NiCo-EPT electrodes at a various current densities in the range of 10 to 100 mA cm^{-2}.

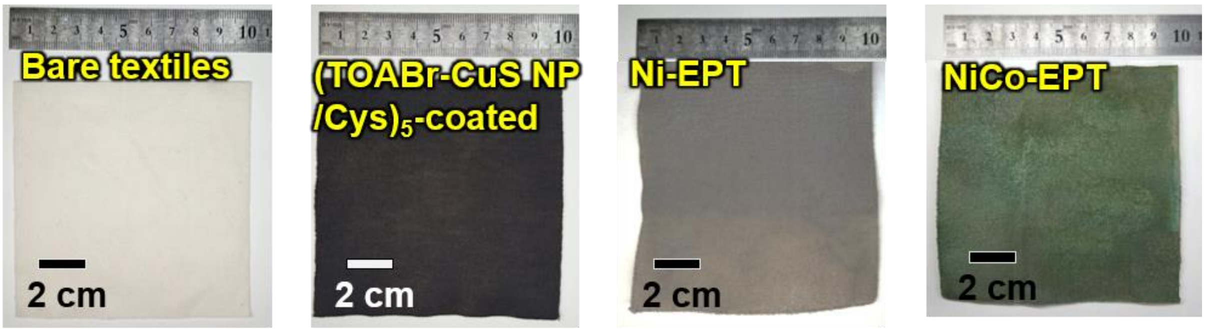

Figure S29. Photographic images of bare textiles, (TOABr-CuS NP/Cys)$_5$-coated textiles, Ni-EPT, and NiCo-EPT in a large-scale of 10 x 10 cm^2.

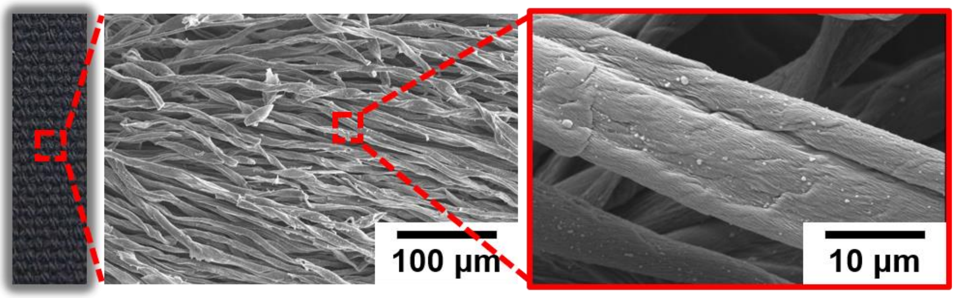

Figure S30. Photographic and planar FE-SEM images of CT electrodes**.**

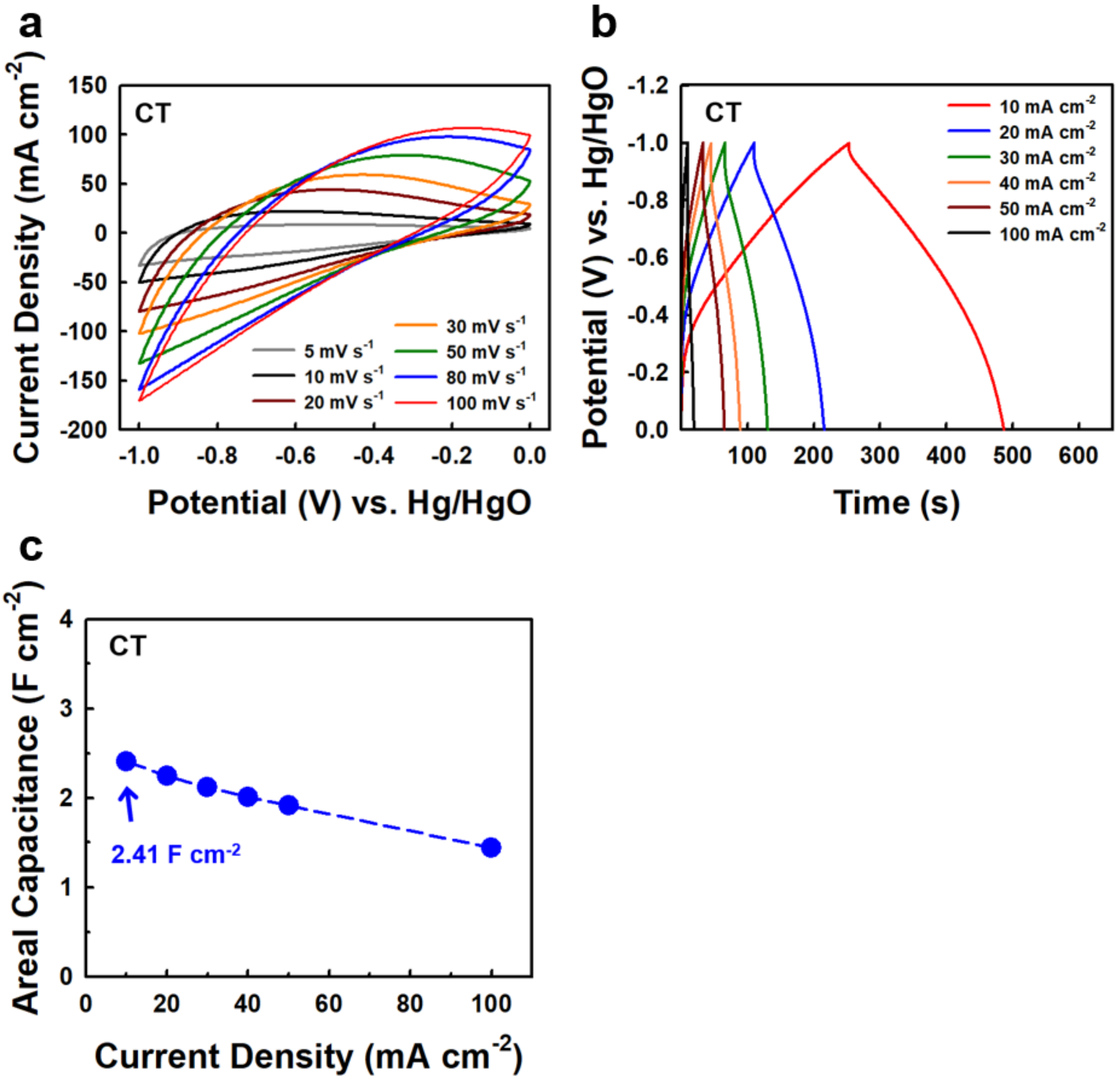

Figure S31. Electrochemical performances of CT electrodes in three-electrode system. a) CV and b) GCD curves at various scan rates and current densities. c) Areal capacitances as a function of the current density (10-100 mA cm^{-2}).

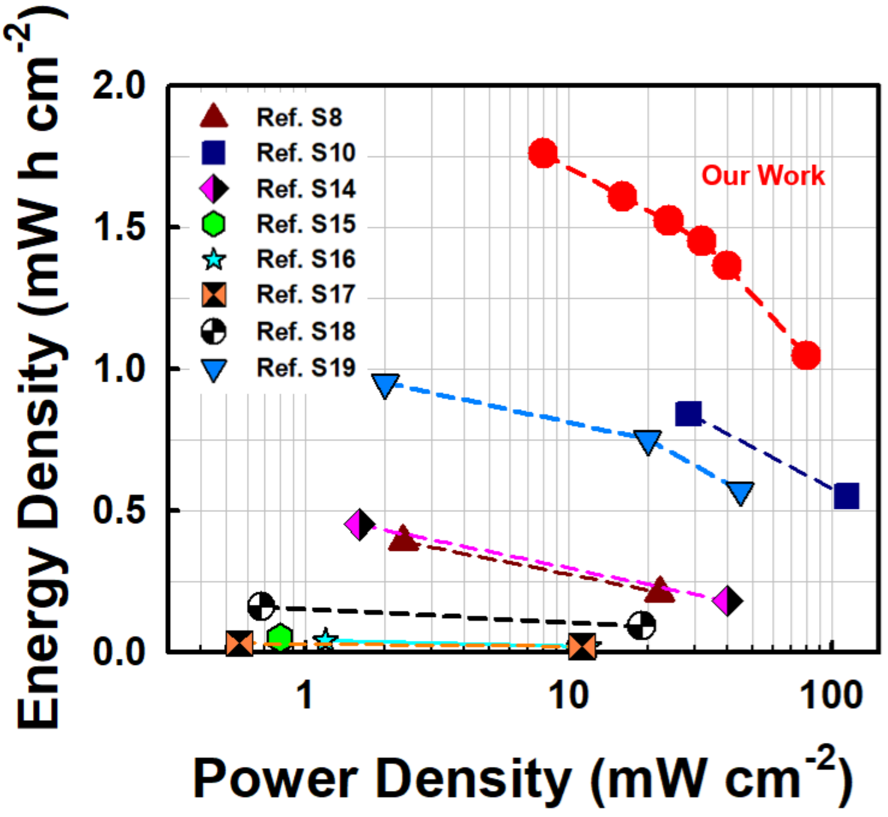

Figure S32. Ragone plots of the AFPs (i.e., NiCo-EPT//CT) compared with previous reported references based on 3D substrates.

Table S1. Performance comparison of pseudocapacitive material-based electrodes on textile substrate.

Active Materials	Method	Substrate	Areal Capacitance	Specific Capacitance	Ref.
NiCo-LDH	Electroplating	Cotton Textile	1-stack 12.2 F cm^{-2} (@ 10 mA cm^{-2}) 3-stack 28.8 F cm^{-2} (@ 30 mA cm^{-2})	1-stack 2383 F g^{-1} (@ 1.95 A g^{-1}) 3-stack 1875 F g^{-1} (@ 1.95 A g^{-1})	**Our work**
NiCo-LDH	Electroplating	PET fiber	0.6 F cm^{-2} (@ 0.6 mA cm^{-2})	2105 F g^{-1} (@ 2 A g^{-1})	[S6]
NiCo-LDH /$FeCo_2O_4$	Electroplating	Carbon Cloth	5.3 F cm^{-2} (@ 2.2 mA cm^{-2})	2426 F g^{-1} (@ 1 A g^{-1})	[S7]
NiCo-LDH /NS [a)]	Hydrothermal /Electroplating	Polyester	1.1 F cm^{-2} (@ 3 mA cm^{-2})	648 F g^{-1} (@ 1.69 A g^{-1})	[S8]
$Co(OH)_2$ /MoCoS	Hydrothermal /Electroplating	Carbon Cloth	1.5 F cm^{-2} (@ 20 mA cm^{-2})	1711 F g^{-1} (@ 22.20 A g^{-1})	[S9]
MnO_2	Electroplating	Carbon Cloth	3.0 F cm^{-2} (@ 3 mA cm^{-2})	304 F g^{-1} (@ 3 A g^{-1})	[S10]
$ZnCo_2O_4$	Dip-coating /Annealing	Carbon Cloth	2.3 F cm^{-2} (@ 0.5 mA cm^{-2})	2034 F g^{-1} (@ 0.45A g^{-1})	[S11]
NiCoO	Hydrothermal	Carbon Cloth	-	1652 F g^{-1} (@ 0.50 A g^{-1})	[S12]
PANI/rGO	Dip-coating /Polymerization	Polyester	1.2 F cm^{-2} (@ 1 mA cm^{-2})	389 F g^{-1} (@ 0.32 A g^{-1})	[S13]

a) NS : Nanosheets.

Table S2. Performance comparison of AFPs based 3D substrates.

Cathode Materials (Substrate)	Anode Materials (Substrate)	Potential	Areal Energy Density (mW h cm^{-2})	Areal Power Density (mW cm^{-2})	Ref.
NiCo-LDH (Cotton Textile)	Carbonized Textiles (CT)	1.6 V	1.77	80	**Our work**
NiCo-LDH /NS [a)] (Polyester)	AC [b)] (Carbon Cloth)	1.6 V	0.39	2.35	[S8]
MnO_2 (Carbon Cloth)	V_2O_5 (Carbon Cloth)	2.0 V	0.84	28.56	[S10]
Co_3O_4 /Graphene (Paper)	r-Bi_2O_3 /Graphene (Paper)	1.6 V	0.45	1.6	[S14]
Co_3O_4 /rGO (CNF[c)])	Graphene Foam	1.6 V	0.05	0.8	[S15]
$NiCo_2O_4$-N[d)] (Carbon Cloth)	PC[e)] (Carbon Cloth)	1.5 V	0.04	1.19	[S16]
$NiCo_2O_4$/C (Paper)	AC (Paper)	1.5 V	0.03	0.56	[S17]
NiCo-LDH (Ni Foam)	AC (Ni Foam)	1.5 V	0.16	0.68	[S18]
$Ni(OH)_2$[)] (Ni Foam)	rGO (Ni Foam)	1.5 V	0.95	2.01	[S19]

References

[S1] O. P. Watts, *Trans. Am. Electrochem. Soc.* **1916**, *29*, 395.

[S2] V. Khomenko, E. Raymundo-Piñero, F. Béguin, *J. Power Sources* **2006**, *153*, 183.

[S3] D. A. Buttry, *Advances in Electroanalytical Chemistry: Applications of the QCM to Electrochemistry*, Marcel Dekker, New York **1991**.

[S4] S. Zhang, N. Pan, *Adv. Energy Mater.* **2015**, *5*, 1401401.

[S5] S. Zhao, Y. Li, H. Yin, Z. Liu, E. Luan, F. Zhao, Z. Tang, S. Liu, *Sci. Adv.* **2015**, *1*, e1500372.

[S6] G. Nagaraju, G. S. R. Raju, Y. H. Ko, J. S. Yu, *Nanoscale* **2016**, *8*, 812.

[S7] X. He, R. Li, J. Liu, Q. Liu, R. chen, D. Song, J. Wang, *Chem. Eng. J.* **2018**, *334*, 1573.

[S8] G. Nagaraju, S. C. Sekhar, L. K. Bharat, J. S. Yu, *ACS Nano* **2017**, *11*, 10860.

[S9] S. J. Patil, D. Lee, *J. Mater. Chem. A* **2018**, *6*, 9592.

[S10] Z. Huang, Y. Song, D. Feng, Z. Sun, X. Sun, X. Liu, *ACS Nano* **2018**, *12*, 3557.

[S11] D. Kong, Y. Wang, S. Huang, J. Hu, Y. V. Lim, B. Liu, S. Fan, Y. Shi, H. Y. Yang, *Energy Storage Mater.* **2019**, *23*, 653.

[S12] T. Wang, Y. Wang, J. Lei, K.-J. Chen, H. Wang, *Exploration* **2021**, *1*, 20210178.

[S13] X. Li, L. Yuan, R. Liu, H. He, J. Hao, Y. Lu, Y. Wang, G. Liang, G. Yuan, Z. Guo, *Adv. Energy Mater.* **2021**, *11*, 2003010.

[S14] R. Liu, L. Ma, G. Niu, X. Li, E. Li, Y. Bai, G. Yuan, *Adv. Funct. Mater.* **2017**, *27*, 1701635.

[S15] J. Huang, Y. Xiao, Z. Peng, Y. Xu, L. Li, L. Tan, K. Yuan, Y. Chen, *Adv. Sci.* **2019**, *6*, 1900107.

[S16] X. Liu, W. Zang, C. Guan, L. Zhang, Y. Qian, A. M. Elshahawy, D. Zhao, S. J. Pennycook, J. Wang, *ACS Energy Lett.* **2018**, *3*, 2462.

[S17] G. Yang, S. Park, *Electrochim. Acta* **2018**, *285*, 405.

[S18] Y. Zhang, H. Hu, Z. Wang, B. Luo, W. Xing, L. Li, Z. Yan, L. Wang, *Nano Energy* **2020**, *68*, 104226.

[S19] D. Shi, L. Zhang, X. Yin, T. J. Huang, H. Gong, *J. Mater. Chem. A* **2016**, *4*, 12144.
